# Potential ash impact from Antarctic volcanoes: Insights from Deception Island’s most recent eruption

**DOI:** 10.1038/s41598-017-16630-9

**Published:** 2017-11-28

**Authors:** A. Geyer, A. Marti, S. Giralt, A. Folch

**Affiliations:** 1 Institute of Earth Sciences Jaume Almera (ICTJA-CSIC), Lluis Solé i Sabaris s/n, 08028 Barcelona, Spain; 2 Barcelona Supercomputing Center (BSC), Jordi Girona 29, 08034 Barcelona, Spain

## Abstract

Ash emitted during explosive volcanic eruptions may disperse over vast areas of the globe posing a threat to human health and infrastructures and causing significant disruption to air traffic. In Antarctica, at least five volcanoes have reported historic activity. However, no attention has been paid to the potential socio-economic and environmental consequences of an ash-forming eruption occurring at high southern latitudes. This work shows how ash from Antarctic volcanoes may pose a higher threat than previously believed. As a case study, we evaluate the potential impacts of ash for a given eruption scenario from Deception Island, one of the most active volcanoes in Antarctica. Numerical simulations using the novel MMB-MONARCH-ASH model demonstrate that volcanic ash emitted from Antarctic volcanoes could potentially encircle the globe, leading to significant consequences for global aviation safety. Results obtained recall the need for performing proper hazard assessment on Antarctic volcanoes, and are crucial for understanding the patterns of ash distribution at high southern latitudes with strong implications for tephrostratigraphy, which is pivotal to synchronize palaeoclimatic records.

## Introduction

Explosive volcanic eruptions pose proximal hazards by tephra fallout and can disperse fine ash and volcanic aerosols over vast areas of the globe thereby generating a threat to human health and infrastructures and causing long-range air traffic disruptions. Several volcanic events that have occurred in recent years, including the 2010 Eyjafjallajökull^1^ (Iceland), 2011 Grímsvötn^2^ (Iceland) and Cordón Caulle^3^ (Chile) eruptions, have led to large economic losses for the aviation industry and its stakeholders, demonstrating the global extent of this phenomenon. For instance, the April 14^th^ 2010 Eyjafjallajökull eruption (Iceland) affected air traffic across western and northern Europe over several days^4^ leading to global GDP (gross domestic product) losses of 4.7 billion US dollars. This figure includes net airline industry and destination losses, along with general productivity losses^5,6^.

**Figure 1:**
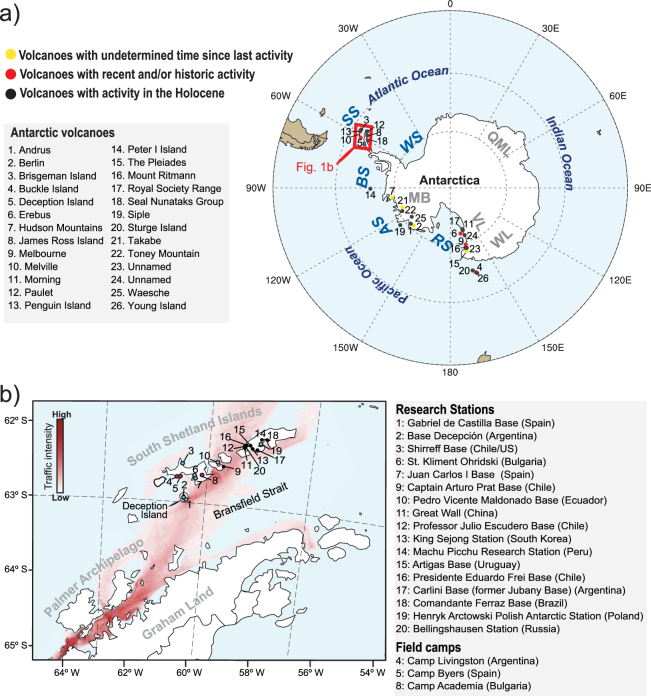
(**a**) Location of Antarctic volcanoes listed in Table S1.1. AS: Amundsen Sea; BS: Bellinghausen Sea; QML: Queen Maud Land; MB: Marie Byrd Land; RS: Ross Sea; SS: Scotia Sea; VL: Victoria Land; WS: Weddell Sea; WL: Wilkes Land. (**b**) Location of year-round (black dots) and temporary (only austral summer, blue dots) research stations nearby Deception Island. Red dots correspond to temporary field camps. The intensity of vessel traffic in the touristic season 2012/13 is also indicated (source: Bender, *et al*.^37^). This figure was generated with NCAR Command Language (NCL) version 6.1.2 (Boulder, Colorado: UCAR/NCAR/CISL/TDD. http://dx.doi.org/10.5065/D6WD3XH5). Final layout was obtained with Adobe Illustrator CC 2015.3.1 (Copyright © 1987–2016 Adobe Systems Incorporated and its licensors).

Of the dozens of volcanoes located in Antarctica, at least nine (Berlin, Buckle Island, Deception Island, Erebus, Hudson Mountains, Melbourne, Penguin Island, Takahe, and The Pleiades) are known to be active and five of them, all stratovolcanoes, have reported frequent volcanic activity in historical times (Fig. 1a) (Table S1.1)(Global Volcanism Program, http://www.volcano.si.edu). To date, the potential risks, at regional and global scales, related to high southern latitude eruptions have never been assessed, albeit damage of Antarctic scientific stations due to volcanic hazards has been repeatedly reported in the past^7,8^. Over the last decades, scientific activity and tourism in Antarctica have augmented considerably—especially at the South Shetland Islands and the Antarctic Peninsula, where the exposure of population and infrastructures to volcanic risk has significantly increased. For example, the islands of Deception and Livingston (South Shetland archipelago) host 5 research stations and 3 summer field camps, whereas Greenwich and King George islands, located in the same archipelago, are home to 10 all-year and 2 temporary research stations (Fig. 1b). Additionally, the Palmer Archipelago and the northwestern coast of Graham Land have become important touristic destinations exceeding 30,000 visitors per year (IAATO, https://iaato.org/), drastically increasing ship traffic during the touristic season (Fig. 1b). Therefore, hazards posed by Antarctic eruptions, especially those related to volcanic ash, need to be properly addressed despite the little interest received so far.

The frequency of explosive ash-forming eruptions happening at high southern latitudes is uncertain. However, considering that basaltic effusive activity may easily become explosive if the rising magma interacts with sea water, snow or ice, the occurrence of moderate to highly explosive eruptions should not be disregarded^9^. This paper analyzes the potential impacts of ash dispersal and fallout from Antarctic volcanoes by focusing on Deception island as a case study, an active volcano with several tens of eruptions in the last 10,000 years^8,10^. We demonstrate here that ash from high southern latitude volcanoes may pose a threat higher than previously believed. Results obtained highlight how ash clouds entrapped in circumpolar upper-level winds clearly have the potential to reach lower latitudes and disrupt Austral hemisphere air traffic. In addition, ash fall out may also cause important regional impacts, not only on the scientific research stations and summer field camps in the area, but also on touristic vessels operating in the region. The outcomes of the present study draw attention to the need to perform dedicated hazard assessments on active Antarctic volcanoes, and are crucial to understanding ash distribution patterns at high southern latitudes. This latter aspect has obvious implications for tephrostratigraphic and chronologic studies that provide valuable isochrones to synchronize paleoclimate records.

**Figure 2:**
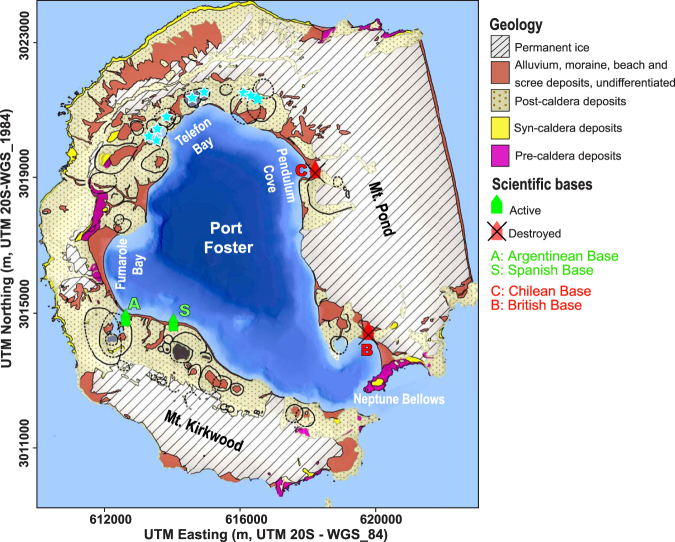
Simplified geological map of DI (modified from Martí *et al*.^38^). Blue stars show the location of the 1970 eruptive centers (data obtained from Spatial Data Infrastructure for Deception Island SIMAC, Torrecillas *et al*.^39^). The site of the remains of the Chilean and British Scientific Bases destroyed by the most recent eruptions, as well as the currently active Argentinean and Spanish Scientific Bases, are also indicated. Black solid and dashed lines delimit visible and inferred post-caldera volcanic craters, respectively. This figure was generated with QGIS software version 2.18 Las Palmas (available at: www.qgis.org). Final layout was obtained with Adobe Illustrator CC 2015.3.1 (Copyright © 1987–2016 Adobe Systems Incorporated and its licensors).

Deception Island (DI), located at the spreading center of the Bransfield Strait marginal basin, consists of a horse-shoe-shaped composite volcanic system truncated by the formation of a collapse caldera represented as a sea-flooded depression known as Port Foster^11^ (Fig. 2). The tephra record from Deception and neighboring islands, reveals over 30 post-caldera Holocene eruptions. However, considerably more eruptions are assumed to have occurred^12^. Indeed, over 50 relatively well-preserved craters and eruptive vents scattered across the island, can be reconstructed and mapped (Fig. 2). The eruption record for Deception Island since the 19^th^ century, reveals periods of high activity (1818–1828, 1906–1912) followed by decades of dormancy (e.g. 1912–1967)^7,12,13^. The unrest episodes recorded in 1992, 1999^14^ and 2014–2015^15^ demonstrate that the volcanic system is still active and may be cause of concern in the future. During the most recent explosive eruptions that occurred in 1967, 1969 and 1970, ash fall and lahars destroyed or severely damaged the scientific bases operating on the island at that time^4^.

Historical volcanism at DI, mostly classified as Volcanic Explosivity Index (VEI) 2–3, involves small to moderate-volume monogenetic eruptions (<0.1 km^3^) with eruptive columns rising up to 10 km in height^9,10,16^. Based on the analysis of past eruptions, the most worrying hazard during a volcanic eruption on DI is ash fall^8,10^. Due to the strong winds and the low altitude of the tropopause in the area (8–10 km)^17^, ash fall deposits are rapidly dispersed^18–20^. In fact, the common presence of DI tephra in lacustrine cores of neighboring islands, marine sediments of the Bransfield Strait and Scotia Sea (>800 km distance), and even in South Pole ice cores, suggests that some post-caldera eruptions may have been significantly more violent than those experienced in recent centuries. These events would have involved eruptive columns exceeding 20 km in height with much larger volumes of magma^17,18,21^. At DI, variations in the degree of explosivity can be explained either by eventual interactions with sea water, underground aquifers, or glacier water with the rising or erupting magma^9^, or by the presence of highly explosive dacitic to rhyolitic magmas such as the ones observed in Cross Hill^11^.

In order to evaluate the significance and impact from ash emitted during a regular eruption at Deception Island, we performed different sets of simulations using the NMMB-MONARCH-ASH^22^ meteorological and atmospheric dispersion model at regional and global scales for a scenario with similar characteristics to the most recent eruption that occurred in 1970^9^. Regional-scale simulations serve to constrain the expected fallout ranges and deposit thicknesses whereas the global-scale ones aim at assessing potential long-range impacts on air traffic. Additionally, to evaluate the ash dispersion dependency on the source location and magnitude (expressed in terms of different column heights), we compared the dispersion results from the DI scenario with: i) those of a similar set-up but where the source is located at higher latitudes —more specifically, at a position equivalent to Mt. Erebus (77.5°S and 167°E)(Fig. 1a); and ii) those from an eruptive event with ± 50% variation at the height of the eruptive column.

**Figure 3:**
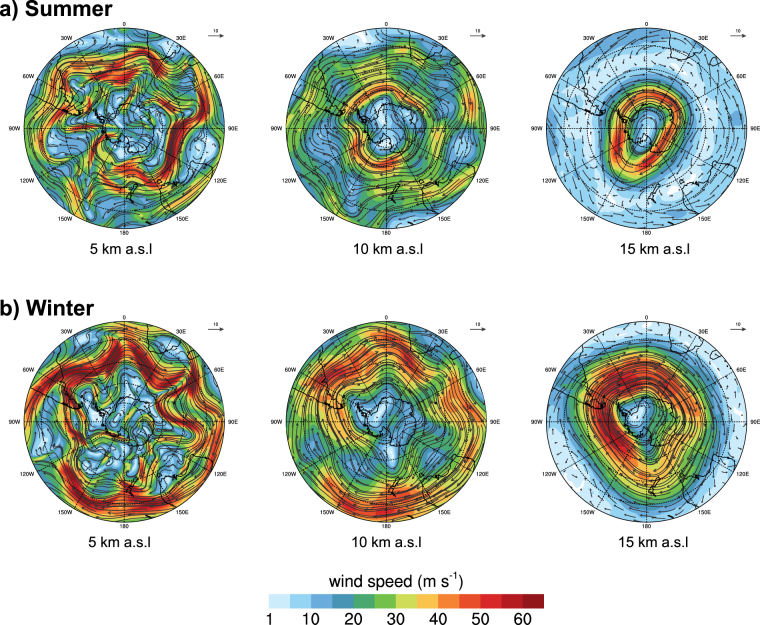
NMMB/BSC-ASH meteorological results over the South Pole during the summer (**a**), and winter (**b**) seasons. Plots show wind vectors and velocity contours (in m∙s^−1^) at 5, 10 and 15 km a.s.l., roughly corresponding to mid-troposphere, tropopause, and stratosphere respectively. For details on model configuration see Table S1.2. This figure was generated with NCAR Command Language (NCL) version 6.1.2 (Boulder, Colorado: UCAR/NCAR/CISL/TDD. http://dx.doi.org/10.5065/D6WD3XH5). Final layout was obtained with Adobe Illustrator CC 2015.3.1 (Copyright © 1987–2016 Adobe Systems Incorporated and its licensors).

**Figure 4:**
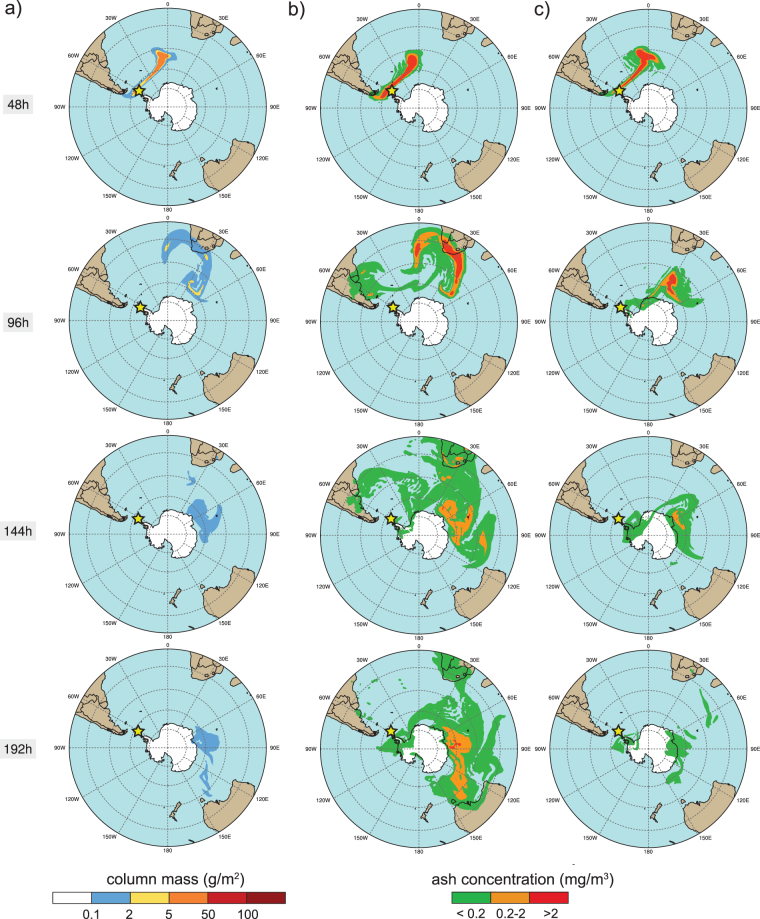
Global-scale NMMB/BSC-ASH model results for the austral summer period at different time instants (2, 4, 6, and 8 days after the eruption start from top to bottom, respectively). An eruptive column of 10 km height was considered for the 1970-like scenario to simulate: (**a**) the total column mass loading (in g∙m^−2^), (**b**) the concentration of ash at Flight Level FL050 (in mg∙m^−3^), and (**c**) the concentration at FL250. Safe ash concentration thresholds are shown (red concentration contours illustrate “No Fly” zones). The yellow star indicates the location of Deception Island. This figure was generated with NCAR Command Language (NCL) version 6.1.2 (Boulder, Colorado: UCAR/NCAR/CISL/TDD. http://dx.doi.org/10.5065/D6WD3XH5). Final layout was obtained with Adobe Illustrator CC 2015.3.1 (Copyright © 1987–2016 Adobe Systems Incorporated and its licensors).

**Figure 5:**
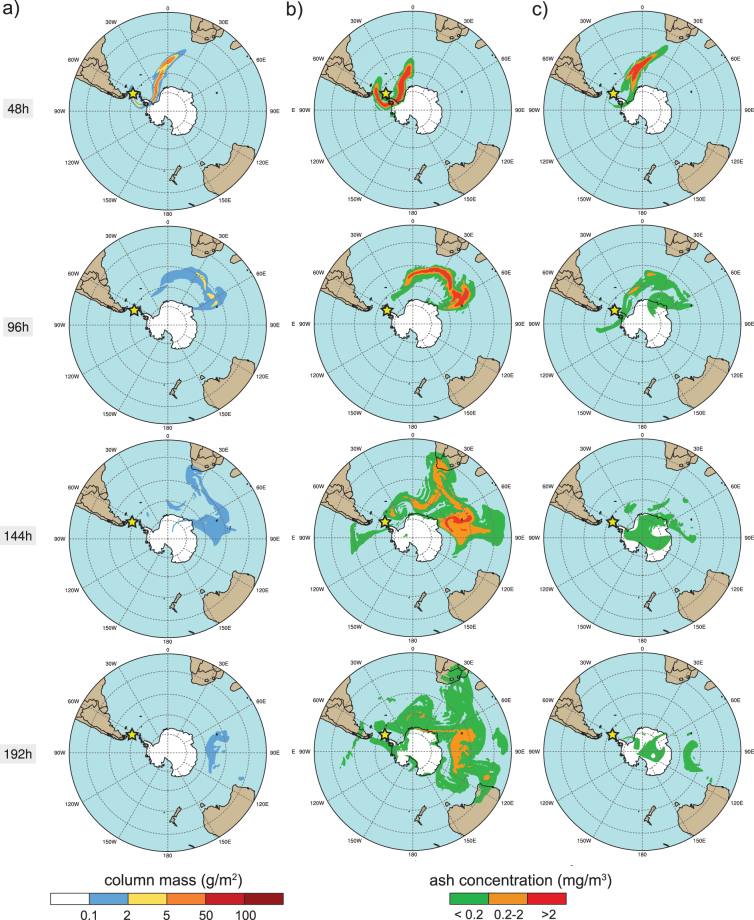
Global-scale NMMB/BSC-ASH model results for the austral winter period at different time instants (2, 4, 6, and 8 days after the eruption start from top to bottom, respectively). An eruptive column of 10 km height was considered for the 1970-like scenario to simulate: (**a**) the total column mass loading (in g∙m^−2^), (**b**) the concentration of ash at Flight Level FL050 (in mg∙m^−3^), and (**c**) the concentration at FL250. Safe ash concentration thresholds are shown (red concentration contours illustrate “No Fly” zones). The yellow star indicates the location of Deception Island. This figure was generated with NCAR Command Language (NCL) version 6.1.2 (Boulder, Colorado: UCAR/NCAR/CISL/TDD. http://dx.doi.org/10.5065/D6WD3XH5). Final layout was obtained with Adobe Illustrator CC 2015.3.1 (Copyright © 1987–2016 Adobe Systems Incorporated and its licensors).

The seasonal climate variability of Antarctica is mainly caused by the Southern Annular Mode (SAM). Changes in this mode explain up to 30% of the deseasonalized variability in both geopotential and winds^23^. For this reason, each set of simulations considers randomly selected meteorological situations (to avoid possible subjective bias in the date selection) representative of the Antarctic summer (1982, S-82) and winter periods (1995, W-95). The chosen periods have been verified to be within the average winter and summer climate conditions in Antarctica based on the most complete databank of Antarctic temperature and pressure data (http://cdiac.esd.ornl.gov/epubs/ndp/ndp032/ndp032.html). Furthermore, two additional simulation sets were performed that coincided with the unrest episodes recorded in 1992 (S-92) and 1999 (S-99). The summer period is especially interesting because it is over the course of this season that tourists and scientific researchers are present in Antarctica, with a corresponding increase in the number of flights in the Southern Hemisphere due to holiday movements. Results are presented in Figs 3 to 5 and in the Supplementary materials S1 (S-82/W-95) and S2 (S-92/S-99). For each of the selected periods of time, the meteorological variables used in the model were averaged in order to obtain the predominant and most representative climate conditions.

## Results

### Meteorological conditions

Figure 3 shows NMMB-MONARCH-ASH model meteorological results over the South Pole during the two selected seasons (S-82/W-95). A persistent large-scale clock-wise circulation around an upper-level low-pressure zone located close to the Pole is clearly visible at any time. The polar vortex extends up to the stratosphere, with global-scale circulation covering latitudes from 70° up to 50° depending on the period. At these stratospheric levels, the resulting polar jet stream is very intense (wind speeds over 60 m/s), widening notably during the winter (Fig. 3b) and narrowing during the summer (Fig. 3a). In the particular case of the summer situation, the jet stream remains confined at around 65° latitude. This is also true close to the tropopause (Fig. S1.2-10 km height plots), where Rossby waves start to form and large-scale wind meandering appears. Finally, at mid-tropospheric levels (Fig. S1.1 and S1.4: 5 km height plots), the meteorological situations show lesser seasonal dependency and are characterized by a breaking of the jet stream and a pronunciation of the meanders reaching much lower latitudes. These well-known synoptic situations have implications on tephra dispersal patterns and anticipate distinct behaviors depending on the volcano location and eruption column height. On the one hand, low plumes (<10 km) from high-latitude (>70°) volcanoes are likely to be confined within the less-intense-winds zone encircled by the jet stream, i.e. displaying no transcontinental dispersal. However, this may not be the case for higher plumes from these volcanoes (Fig. S1.3 and S1.6: 15 km height plots), which could be advected towards the continental periphery and then entrapped by the jet stream before ash settles on the ground. On the other hand, ash released at any height from lower-latitude volcanoes (e.g. Deception Island) is more likely to encircle the globe and reach sub-polar latitudes by meandering advection. Meteorological model results for all climatological periods are presented in detail in the Supplementary material 1 (Fig. S1.1–S1.6) and 2 (Fig. S2.1–S2.2).

### Long-range ash dispersal

We performed global and regional-scale ash dispersal simulations for the DI 1970-like scenario under different climatically-representative meteorological situations. Figures 4 and 5 show global-scale model results for the summer and winter seasons, respectively. At a global scale, moderate to high values of ash column load (>1 g∙m^−2^) are found up to 4 days (96 h) after the eruption onset (see Figs S1.7–S1.12 and S2.3–S2.4). In all simulations, the highest cloud column mass load values (>100 g∙m^−2^) are limited to the first 48 h after the eruption starts and are mainly found over the Atlantic Ocean, over the Scotia and the Weddell Seas. However, residual amounts of ash (0.1–1 g∙m^−2^) are still present in the atmosphere up to 8 days after the eruption’s onset. Ash concentrations above the flight safety thresholds (0.2–2 mg∙m^−3^, orange and red contours in Figs 4 and 5, S1.13–S1.18, S2.5–S2.6) can be observed over South Africa and, in some cases, also over southern Australia or even over austral Patagonia, confirming the potential threat of this DI eruptive scenario to aviation. Depending on the specific wind conditions, some ash clouds re-enter back to the Antarctic Continent over Queen Maud Land during the first 48 h after the eruption (e.g. Figure 5) or over Wilkes Land (with lower concentrations) after longer times (e.g. 72 h in Fig. S1.12). However, in most cases ash clouds circulate around (latitudes 70°–50°) and away (<50°) from the continent, i.e. leaving no substantial fallout record on the main land, highlighting the possibility that many DI eruptions are not registered in the form of tephra layers in South Pole ice cores.

### Ash fallout

**Figure 6:**
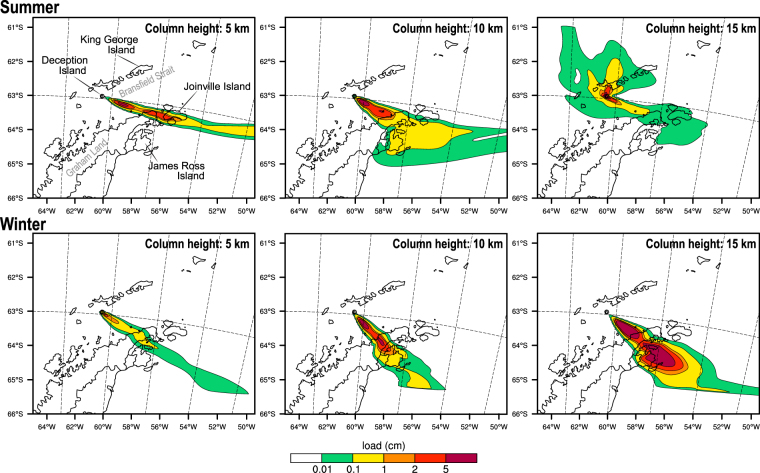
Regional-scale model ground deposit thickness (in cm) for the 1970-like scenario with different column heights of: 5 km (left), 10 km (middle), and 15 km (right). Top and bottom panels show the corresponding ash deposition for the summer and winter periods, respectively. This figure was generated with NCAR Command Language (NCL) version 6.1.2 (Boulder, Colorado: UCAR/NCAR/CISL/TDD. http://dx.doi.org/10.5065/D6WD3XH5). Final layout was obtained with Adobe Illustrator CC 2015.3.1 (Copyright © 1987–2016 Adobe Systems Incorporated and its licensors).

Figure 6 shows the ash fallout deposits for the different meteorological conditions. Understandably, the precise orientations or the deposit axes depend on the regional winds during the selected days, which dispersed coarser particles predominantly to the southeast and southwest of DI. For this particular study, deposits exceeding 1 cm in thickness can be found at distances as far as James Ross Island (>190 km) or beyond Joinville Island (>230 km). In any case, the results highlight the potential for fallout to impact research stations and touristic destinations around Palmer Archipelago and Bransfield Strait locations.

## Discussion and Implications

Although hosting volcanoes with recent activity, the regional and global effects of a potential ash-forming explosive eruption occurring in Antarctica have never before been analyzed. The novel results presented here demonstrate how Antarctic volcanoes may pose a higher threat than previously realized. The potential impact associated with volcanic ash from Antarctic eruptions is mainly contingent on volcano location and eruption column height (*h*). In that context, lower plumes (*h* < 10 km) from high-latitude (>70°) eruptive events are likely to be confined close to the South Pole due to moderated wind zones encircled by the polar jet stream, while higher plumes have a higher potential for transcontinental ash dispersal. However, contrary to this situation, ash from lower-latitude Antarctic volcanoes (e.g. Deception Island) is more likely to encircle the globe, even for moderate size eruptions. In this case, volcanic ash clouds could reach up to tropical latitudes, a vast part of the Atlantic coast of South America, South Africa and/or Oceania. Thus, a wider dispersion of volcanic particles than previously believed may be originated resulting in significant consequences to global aviation safety. For example, Flight Level (FL) ash concentrations resulting from our NMMB-MONARCH-ASH simulations (Figs 4, 5, S1.13–1.18, S2.5–S2.6) at FL050 (~ 1,500 m), FL250 (~7,600 m) and FL350 (~ 10,650 m), clearly show areas over which air traffic would be disrupted due to the presence of ash in the atmosphere (assuming “No Fly” zones with a threshold of 2 mg∙m^−3^). This is true not only in proximity to the South Shetland Islands, i.e. South American airports (over 1,000 km in distance) (e.g. Figure 4), but also in South Africa (over 6,400 km away), affecting international and domestic flying routes, in addition to flights connecting Africa with South America and Australia (e.g. Fig. 5 or Fig S2.5 and S2.6). Similar to events with smaller eruption columns (e.g. *h* = 5 km), dispersal can result in “No Fly” zones affecting all flight routes towards international airports such as Buenos Aires and up to tropical latitudes (<25° S, Tropic of Capricorn) (e.g. Fig. S1.13). For strong eruptive columns (e.g. *h* = 15 km), ash takes longer to settle and areas, including parts of West Antarctica, the Amundsen and Bellinghausen Seas and the South Pole, would still be disrupted by the presence of volcanic ash, even eight days after the onset of the eruption (Fig. S1.15). These results clearly recall the need for further research in the area in order to investigate the potential occurrence of an eruption on DI and to perform a complete hazard assessment for other active Antarctic volcanoes located on West Antarctica and along Victoria Land. Within this framework, a comprehensive statistical analysis concerning the variability of the climate conditions in and around Antarctica, which may control the extent of the volcanic ash impact and related eruption cloud, is recommended. It would be particularly interesting to assess the potential effects of the main climate modes acting in the region to cover all possible climate scenarios (e.g. SAM, El Niño Southern Oscillation) during a future eruption. Lastly, considering the historical volcanic activity in Antarctica, future work would also benefit from an evaluation of the potential volcanic ash impact on climate and air traffic from larger eruptions (VEI > 3).

Recent work has highlighted the wide range of impacts that tropical explosive volcanic eruptions can have on climate modes^24^. These impacts are significant both in time and spatial distribution when the height of the plume reaches the stratosphere. While the main mode of variability of the atmospheric circulation in the Southern Hemisphere extratropics is the SAM^25^, the last 50 years of seasonal and decadal climate variability of Antarctica have been described as a consequence of the interplay between the former climate mode and the El Niño Southern Oscillation (ENSO)^26^. The simultaneous occurrence of positive/negative SAM and La Niña/El Niño events enhance the effects of these main climate modes in Antarctica whereas when positive SAM and El Niño or negative SAM and La Niña conditions prevail, the SAM impacts diminish^27^. The environmental consequences of these climate variations can be clearly observed in sea temperatures, sea ice, glacial melting, and marine ecosystems, among others^28^. However, the effects of explosive volcanic eruptions on these extratropical regions are poorly studied and understood. In the tropics, depending on the ENSO phase and its intensity, the reduction of incoming surface solar radiation in winter resulting from injection in the stratosphere can either initiate or amplify an El Niño event, or shorten the duration of a La Niña event^29^. To date, little is known about what would be the environmental and climate consequences if this eruption took place at high latitudes^30^ or which role might play the reduction of solar radiation on the seasonal, interannual and decadal climate variability of Antarctica. The correct assessment of these impacts might require a multi-model analysis and a comparison with multiple proxy sources^24^.

Finally, understanding how and where volcanic ashes disperse and deposit can provide important information concerning the style, energy and dynamics of past eruptions. The reconstruction and characterization of ash fallout deposits provide valuable information to be used in tephrochronological studies for establishing isochrones used to synchronize palaeoclimatic records. However, even at proximal distances, ash deposits under strong wind dispersal can be very directional and blank limited areas, and therefore be difficult to find. This has to be considered when doing tephrochronological studies in the area. According to our simulations, it is feasible that eruptions occurring in Antarctica do not lead to clear ash deposits on the Antarctic continent or, if observed, they may be of low magnitude.

## Methods

### NMMB-MONARCH-ASH model

NMMB-MONARCH-ASH^31^ is a novel on-line meteorological and atmospheric transport model to simulate the emission, transport and deposition of tephra (ash) particles released from volcanic eruptions. The model predicts ash cloud trajectories, concentration at relevant flight levels, and deposit thicknesses for both regional and global domains. Ash concentration values at FL050 (5,000 feet at nominal pressure) are relevant for airport closure whereas concentrations at FL250 and FL350 (25,000 and 35,000 feet) are critical to airspace closure and to prevent encounters with in-flight airplanes. The on-line coupling of the model allows solving both the meteorological and particle/aerosol transport concurrently and interactively at every time-step. This coupling strategy offers a more realistic representation of the meteorological conditions, improving the current state-of-the-art of tephra dispersal models, especially in situations where meteorological conditions are changing rapidly in time, two-way feedbacks are significant, or distal ash cloud dispersal simulations are required^32^. The model builds on the NMMB/BSC Chemical Transport Model (NMMB/BSC-CTM^33,34^) to represent the transport of volcanic particles. Its meteorological core, the Non-hydrostatic Multiscale Model on a B grid (NMMB^33,35^) allows for nested global-regional atmospheric simulations by using consistent physics and dynamics formulations. The Arakawa B-grid horizontal staggering is applied in the horizontal coordinate employing a rotated latitude-longitude coordinate for regional domains and a latitude-longitude coordinate with polar filtering for global domains. In the vertical, the Lorenz staggering vertical grid is used with a hybrid sigma-pressure coordinate. NMMB-MONARCH-ASH solves the mass balance equation for volcanic ash allowing for different modeling options to account for the characterization of the source term (emissions), the transport of volcanic particles (advection/diffusion), and the particle removal mechanisms (sedimentation/deposition). A description of these options and their governing equations are described in Marti *et al*.^27^.

### Model set-up

Table S1.2 summarizes the NMMB-MONARCH-ASH model configuration for the global and regional domains. The Eruption source parameters (ESP) for the 1970-like scenario were obtained from Pedrazzi *et al*.^9^, who inferred a column height of 10 km and a volume of 0.1 km^3^. The model estimates the mass eruption rate from the column height and atmospheric conditions above the vent based on the parameterization of Bonadonna and Costa^36^. The particle Total Grain Size Distribution (TGSD) was reconstructed from tephra deposits measured at Livingstone island and discretized in 5 bins ranging from 2Φ (0.5 mm) to 7Φ (8 μm) with a linear dependency of particle density on diameters ranging from 1650 to 2800 kg∙m^−3^ (Table S1.3). It should be noted that, due to the lack of sampling in more proximal locations, the resulting TGSD is fine-skewed. As a consequence, we expect simulations to slightly underestimate deposit load and to overestimate far-range concentrations.

## Electronic supplementary material

Supplementary Material

NMMB-MONARCH-ASH simulation during the austral summer period of 1982 for a column height of 5km

NMMB-MONARCH-ASH simulation during the austral summer period of 1982 for a column height of 10km

NMMB-MONARCH-ASH simulation during the austral summer period of 1982 for a column height of 15km
