# Supplementary material for: Potential ash impact from Antarctic volcanoes: Insights from Deception Island’s most recent eruption
Source: Sci Rep. 2017 Nov 28;7:16534. doi: 10.1038/s41598-017-16630-9 (PMC5705727; doi:10.1038/s41598-017-16630-9)
Supplement: Supplementary file 1 — Supplementary Material [file 41598_2017_16630_MOESM1_ESM.pdf]

Potential ash impact from Antarctic volcanoes:

Insights from Deception Island's most recent eruption

A. Geyer[(1)], A. Martí[(2)], S. Giralt[(1)], A. Folch[(2)]

(1) Institute of Earth Sciences Jaume Almera (ICTJA-CSIC), Lluis Solé i Sabaris s/n, 08028 Barcelona, Spain

(2) Barcelona Supercomputing Center (BSC), Jordi Girona 29, 08034 Barcelona, Spain

SUPPLEMENTARY MATERIAL 1

Summer 1982 / Winter 1995

Volcano name	Primary volcano type	Last eruption year	lat (°)	lon (°)
Andrus	Shield(s)	Unknown	-75.8	-132.33
Berlin	Shield(s)	-8350	-76.05	-136
Bridgeman Island	Stratovolcano	Unknown	-62.061	-56.717
Buckle Island	Stratovolcano	1899	-66.78	163.25
Deception Island	Caldera	1970	-63.001	-60.652
Erebus	Stratovolcano	2016	-77.53	167.17
Hudson Mountains	Stratovolcano(es)	-210	-74.33	-99.42
James Ross Island	Shield	Unknown	-64.15	-57.75
Melbourne	Stratovolcano	1892	-74.35	164.7
Melville	Stratovolcano	Unknown	-62.02	-57.67
Morning	Shield	Unknown	-78.5	163.53
Paulet	Pyroclastic cone	Unknown	-63.579	-55.78
Penguin Island	Stratovolcano	1905	-62.1	-57.93
Peter I Island	Shield	Unknown	-68.85	-90.58
Pleiades, The	Stratovolcano	-1050	-72.67	165.5
Rittmann, Mount	Shield	Unknown	-73.45	165.5
Royal Society Range	Pyroclastic cone(s)	Unknown	-78.25	163.33
Seal Nunataks Group	Pyroclastic cone(s)	Unknown	-65.03	-60.05
Siple	Shield	Unknown	-73.43	-126.67
Sturge Island	Stratovolcano	Unknown	-67.4	164.83
Takahe	Shield	-5550	-76.28	-112.08
Toney Mountain	Shield	Unknown	-75.8	-115.83
Unnamed	Pyroclastic cone(s)	Unknown	-73.45	164.58
Unnamed	Submarine	Unknown	-76.83	163
Waesche	Shield(s)	Unknown	-77.17	-126.88
Young Island	Stratovolcano	Unknown	-66.42	162.47

Table S1.1. List of Antarctic volcanoes and last eruptions according to the Global Volcanism Program (last accessed 10/17/2016).

NMMB-MONARCH-ASH CONFIGURATION		
	**Global Run**	**Regional Run**
**Dynamics**	NMMB (180s time-step)	NMMB (10s time-step)
**Physics**	Ferrier microphysics<br>BMJ cumulus scheme<br>MYJ PBL scheme<br>LISS land surface model	
**Aerosols**	5 ash bins	
**Source Term (emissions)**		
Run duration	9 days	
Eruption duration	12 h	
Vertical distribution	Point source	
MER formulation	Degruyter and Bonadonna (2012)[1]	
**Sedimentation model**	Ganser (1993)[2]	
**Run**	**Global Set-up**	**Regional Set-up**
Number of processors	512	256
Domain	Global	Regional
Horizontal resolution	1º x 0.75º	0.052º x 0.037º
Vertical layers	60	
Top of the atmosphere	21 hPa	
Meteorology Boundary Conditions (spatial resolutions)	ECMWF EraInterim Reanalysis (0.75º x 0.75º)	

Table S1.2. NMMB-MONARCH-ASH model configurations. The regional run used a horizontal resolution of 0.052º x 0.037º with a 10s dynamic time-step, while the global domain used a horizontal resolution of 1º x 0.75º with a 180s dynamic time-step.

size<br>(Φ)	diameter<br>(mm)	density<br>(kg m^{-3})	sphericity<br>(-)	mass fraction<br>(%)
2	0.5	1666	0.9	2.92
3	0.25	1950	0.9	30.20
4	0.125	2233	0.9	33.80
5	0.031	2516	0.9	30.02
6	0.01	2800	0.9	3.05

Table S1.3. Particle properties and grain-size distribution employed for all runs.

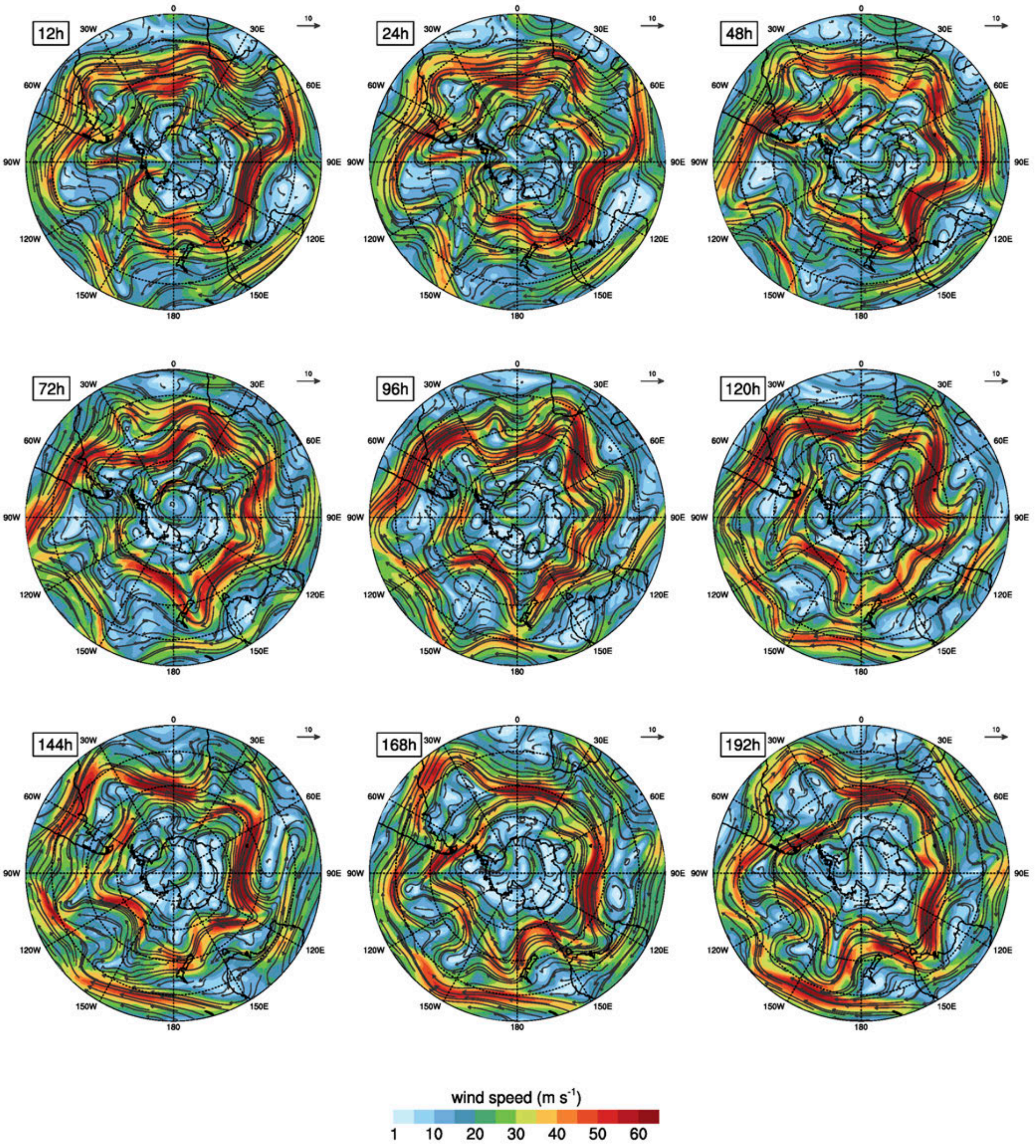

Figure S1.1. NMMB-MONARCH-ASH meteorological model results over the South Pole during the Austral summer period. Plots show wind vectors and velocity contours (in ms^{-1}) at 5 km height during 8 days (192h). This figure was generated with NCAR Command Language (NCL) version 6.1.2 (Boulder, Colorado: UCAR/NCAR/CISL/TDD. http://dx.doi.org/10.5065/D6WD3XH5). Final layout was achieved using Adobe Illustrator CC 2015.3.1 (Copyright © 1987–2016 Adobe Systems Incorporated and its licensors).

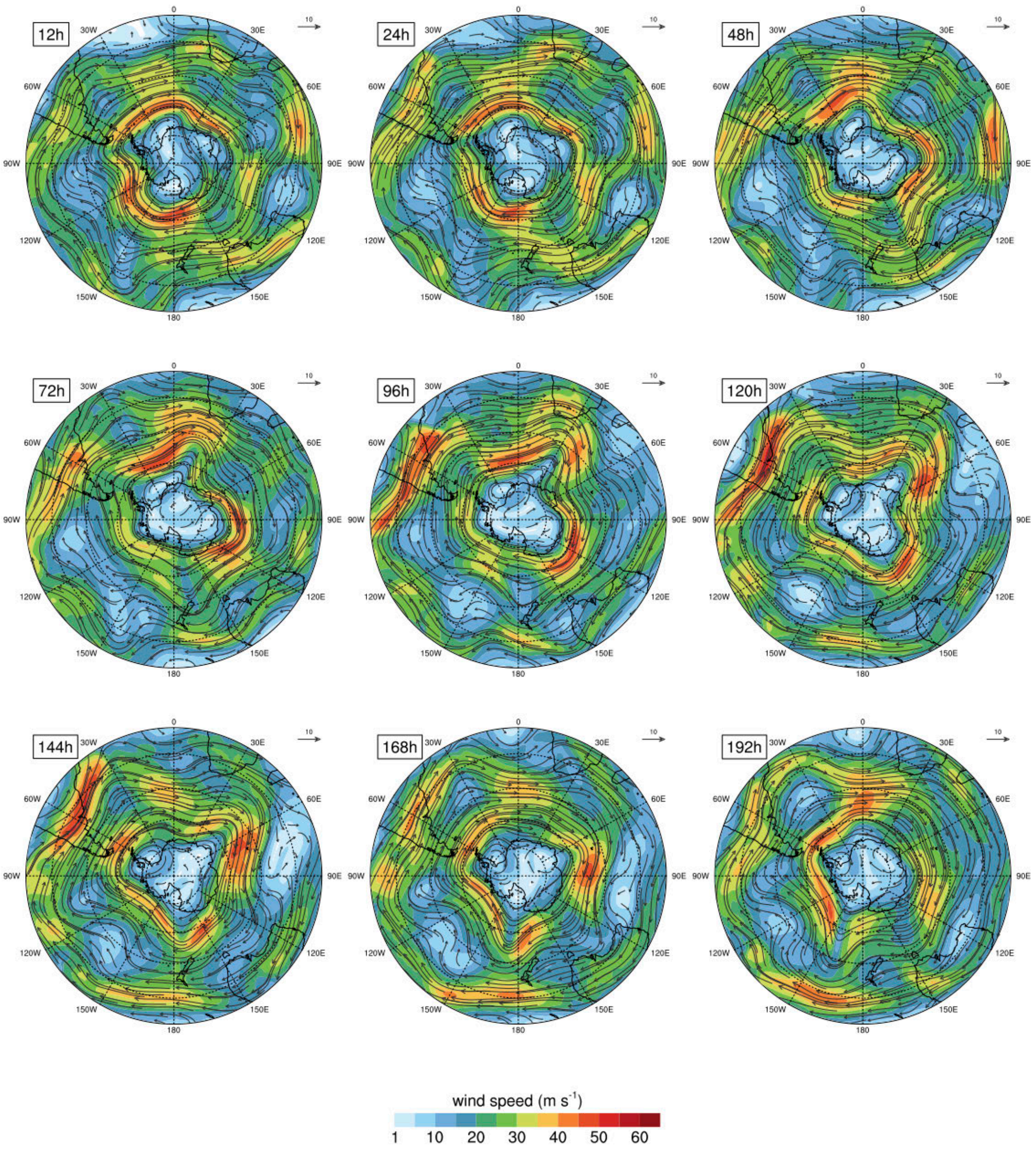

Figure S1.2. NMMB-MONARCH-ASH meteorological model results over the South Pole during the Austral summer period. Plots show wind vectors and velocity contours (in ms^{-1}) at 10 km height during 8 days (192h). This figure was generated with NCAR Command Language (NCL) version 6.1.2 (Boulder, Colorado: UCAR/NCAR/CISL/TDD. http://dx.doi.org/10.5065/D6WD3XH5). Final layout was achieved using Adobe Illustrator CC 2015.3.1 (Copyright © 1987–2016 Adobe Systems Incorporated and its licensors).

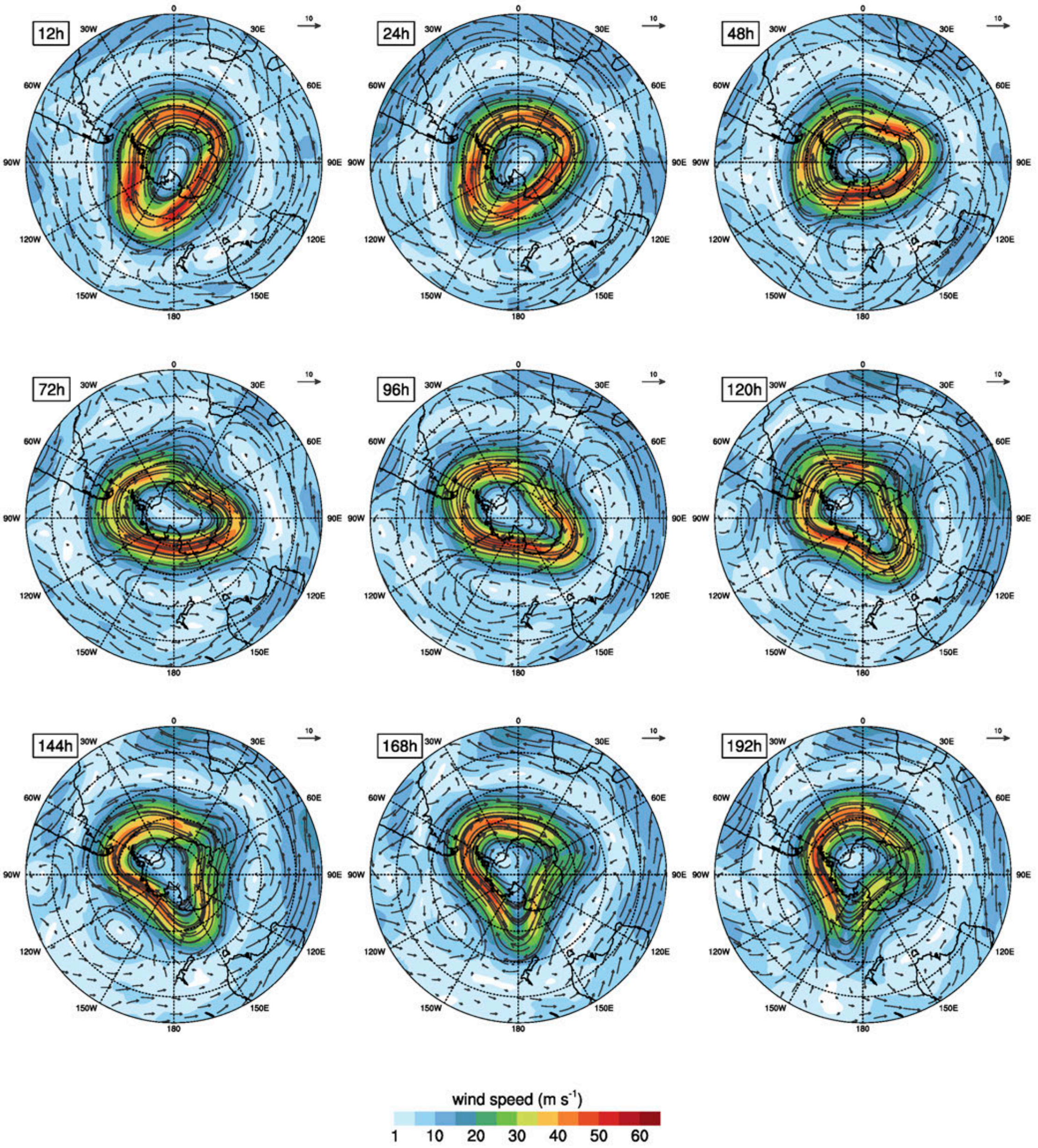

Figure S1.3. NMMB-MONARCH-ASH meteorological model results over the South Pole during the Austral summer period. Plots show wind vectors and velocity contours (in ms^{-1}) at 15 km height during 8 days (192h). This figure was generated with NCAR Command Language (NCL) version 6.1.2 (Boulder, Colorado: UCAR/NCAR/CISL/TDD. http://dx.doi.org/10.5065/D6WD3XH5). Final layout was achieved using Adobe Illustrator CC 2015.3.1 (Copyright © 1987–2016 Adobe Systems Incorporated and its licensors).

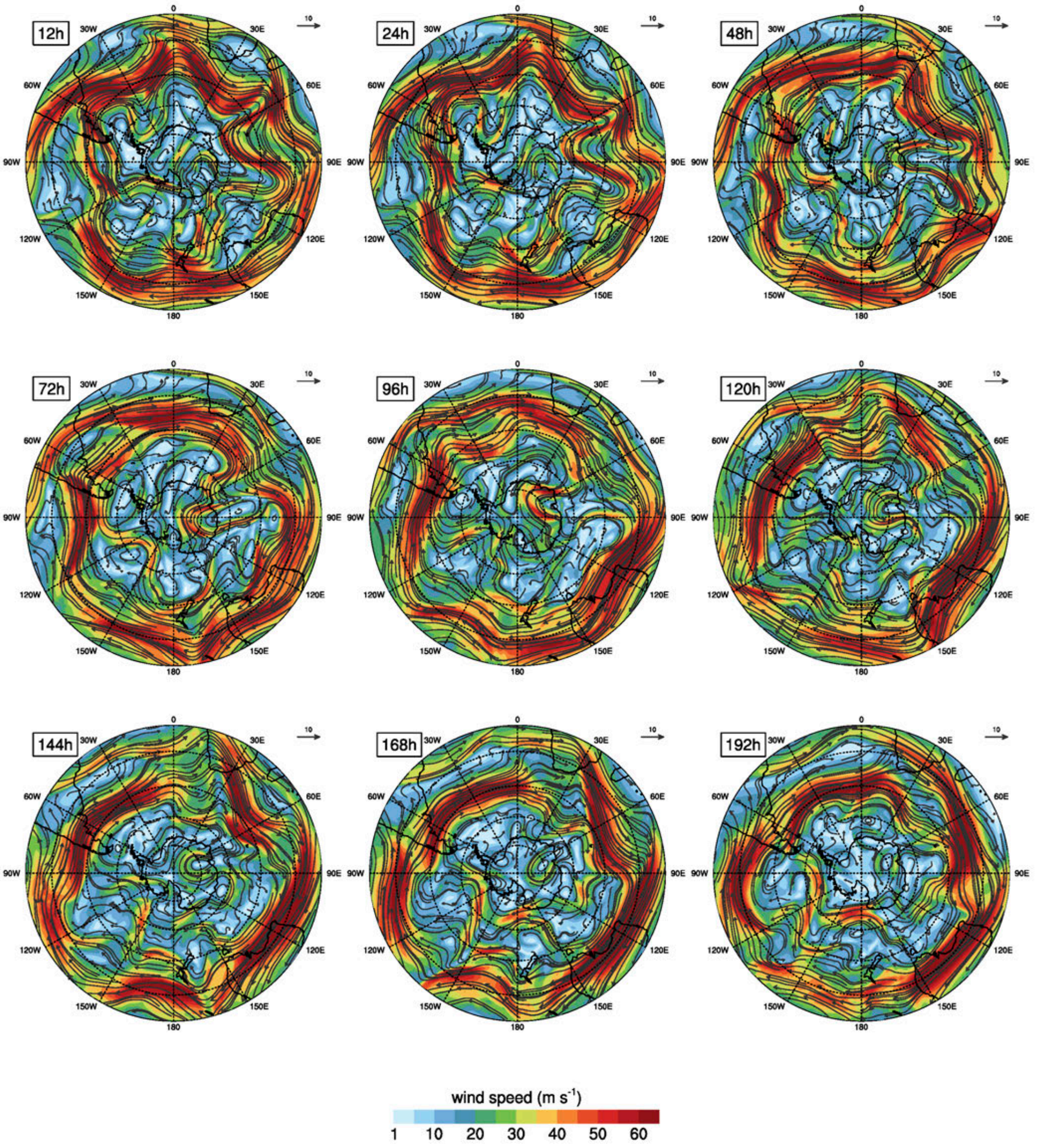

Figure S1.4. NMMB-MONARCH-ASH meteorological model results over the South Pole during the Austral winter period. Plots show wind vectors and velocity contours (in ms^{-1}) at 5 km height during 8 days (192h). This figure was generated with NCAR Command Language (NCL) version 6.1.2 (Boulder, Colorado: UCAR/NCAR/CISL/TDD. http://dx.doi.org/10.5065/D6WD3XH5). Final layout was achieved using Adobe Illustrator CC 2015.3.1 (Copyright © 1987–2016 Adobe Systems Incorporated and its licensors).

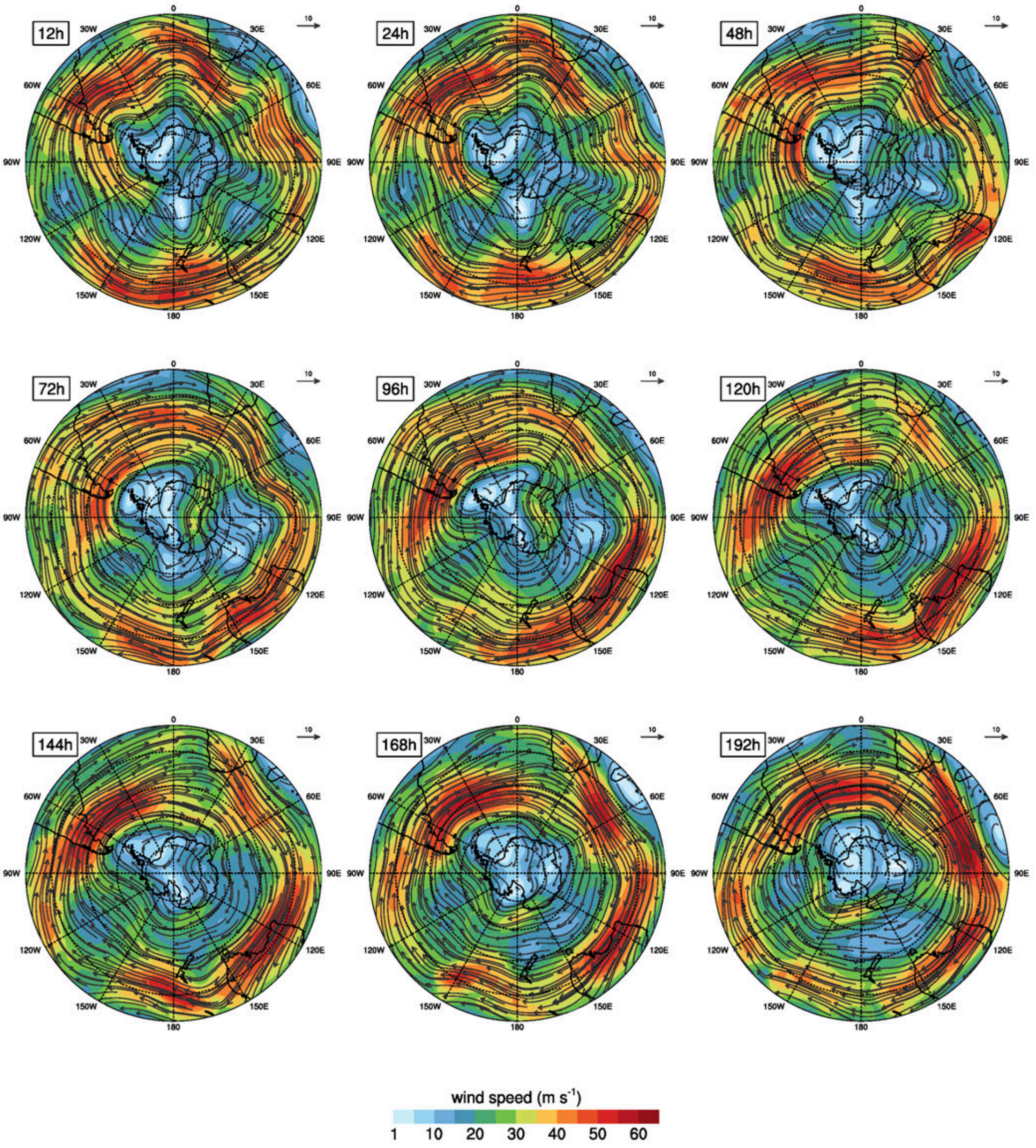

Figure S1.5. NMMB-MONARCH-ASH meteorological model results over the South Pole during the winter period. Plots show wind vectors and velocity contours (in ms^{-1}) at 10 km height during 8 days (192h). This figure was generated with NCAR Command Language (NCL) version 6.1.2 (Boulder, Colorado: UCAR/NCAR/CISL/TDD. http://dx.doi.org/10.5065/D6WD3XH5). Final layout was achieved using Adobe Illustrator CC 2015.3.1 (Copyright © 1987–2016 Adobe Systems Incorporated and its licensors).

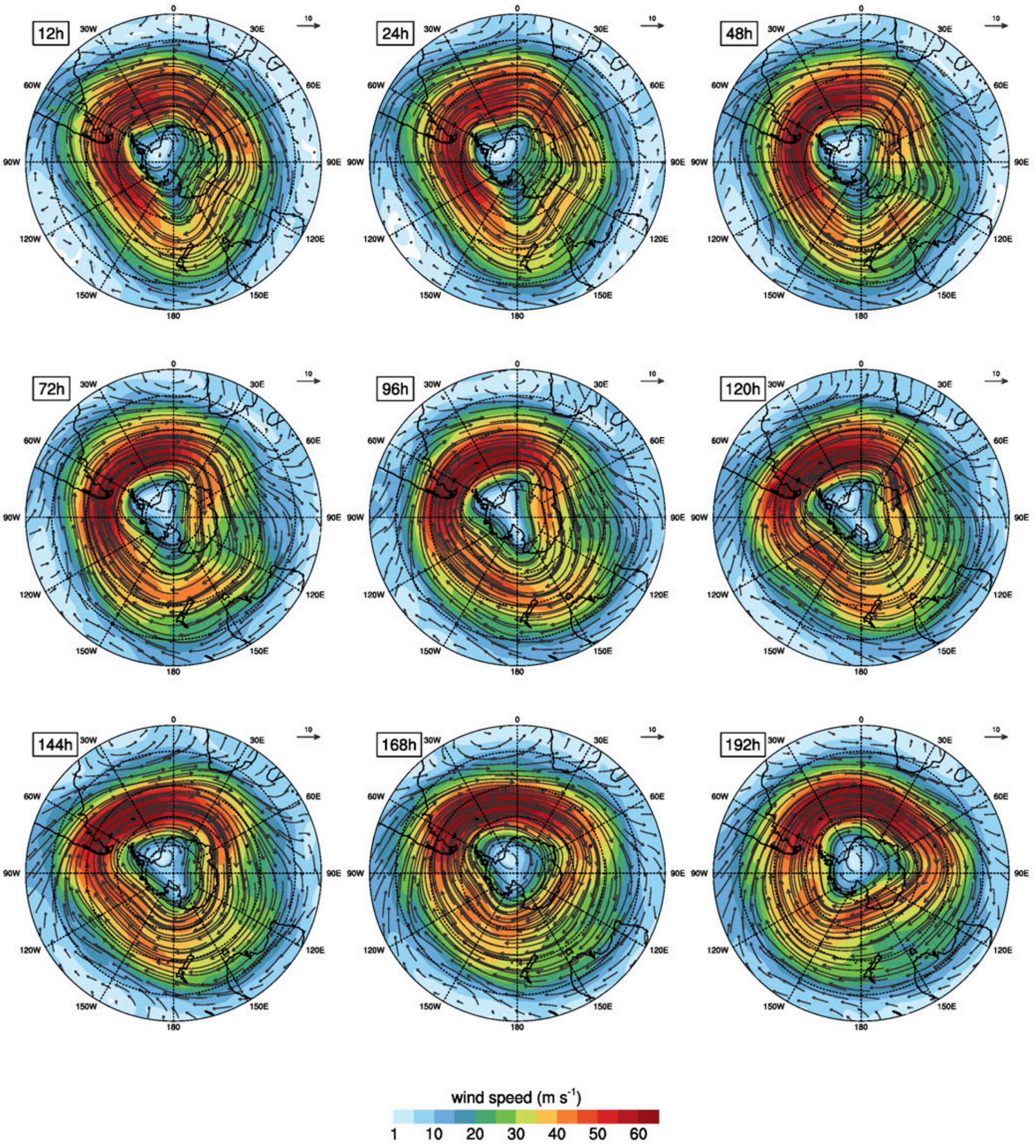

Figure S1.6. NMMB-MONARCH-ASH meteorological model results over the South Pole during the Austral winter period. Plots show wind vectors and velocity contours (in ms^{-1}) at 15 km height during 8 days (192h). This figure was generated with NCAR Command Language (NCL) version 6.1.2 (Boulder, Colorado: UCAR/NCAR/CISL/TDD. http://dx.doi.org/10.5065/D6WD3XH5). Final layout was achieved using Adobe Illustrator CC 2015.3.1 (Copyright © 1987–2016 Adobe Systems Incorporated and its licensors).

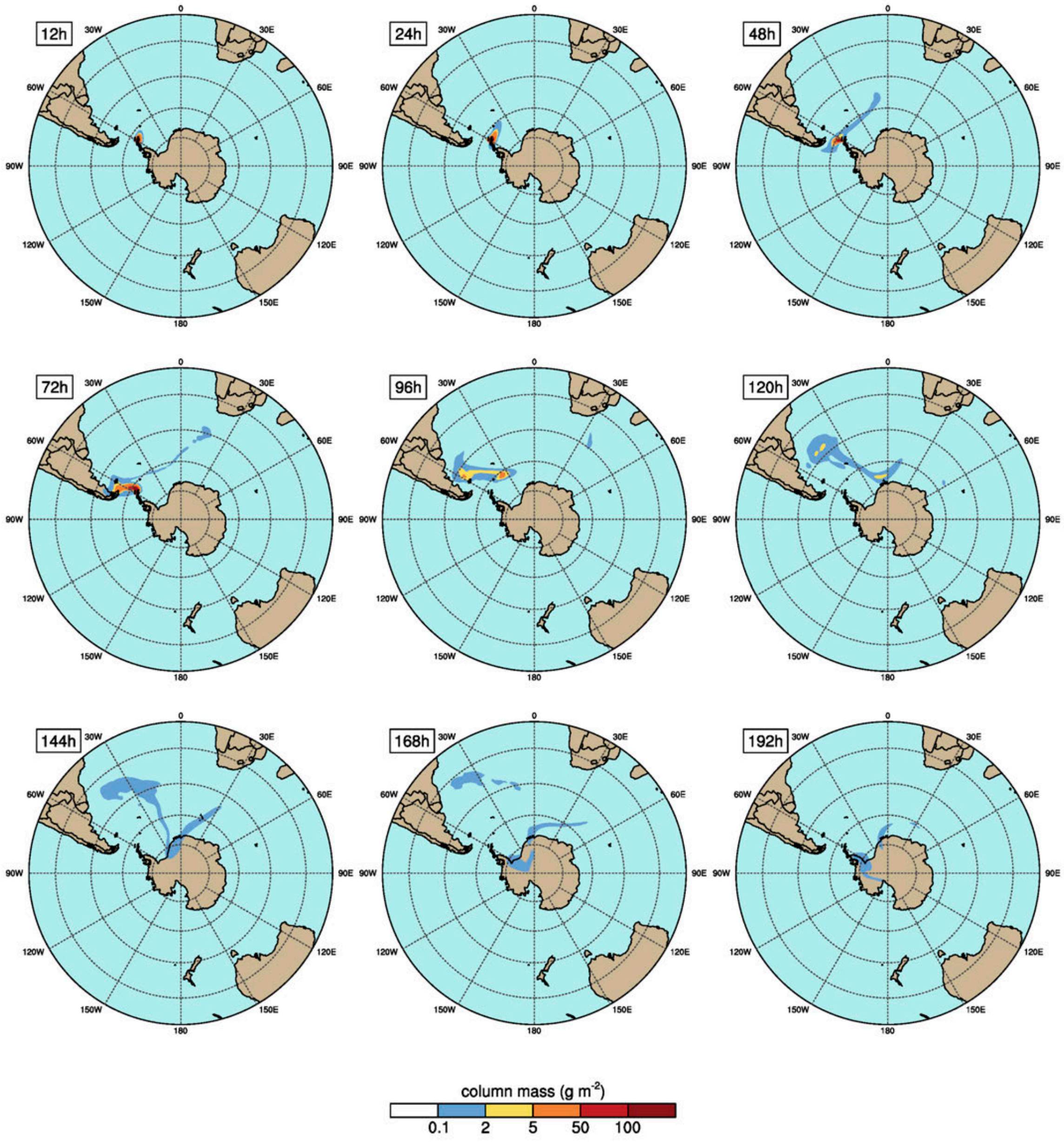

Figure S1.7. NMMB-MONARCH-ASH total ash column mass load (in g m^{-2}) during the Austral summer period at different time instants after the eruption start. Simulation considering an eruption column height of 5 km. This figure was generated with NCAR Command Language (NCL) version 6.1.2 (Boulder, Colorado: UCAR/NCAR/CISL/TDD. http://dx.doi.org/10.5065/D6WD3XH5). Final layout was achieved using Adobe Illustrator CC 2015.3.1 (Copyright © 1987–2016 Adobe Systems Incorporated and its licensors).

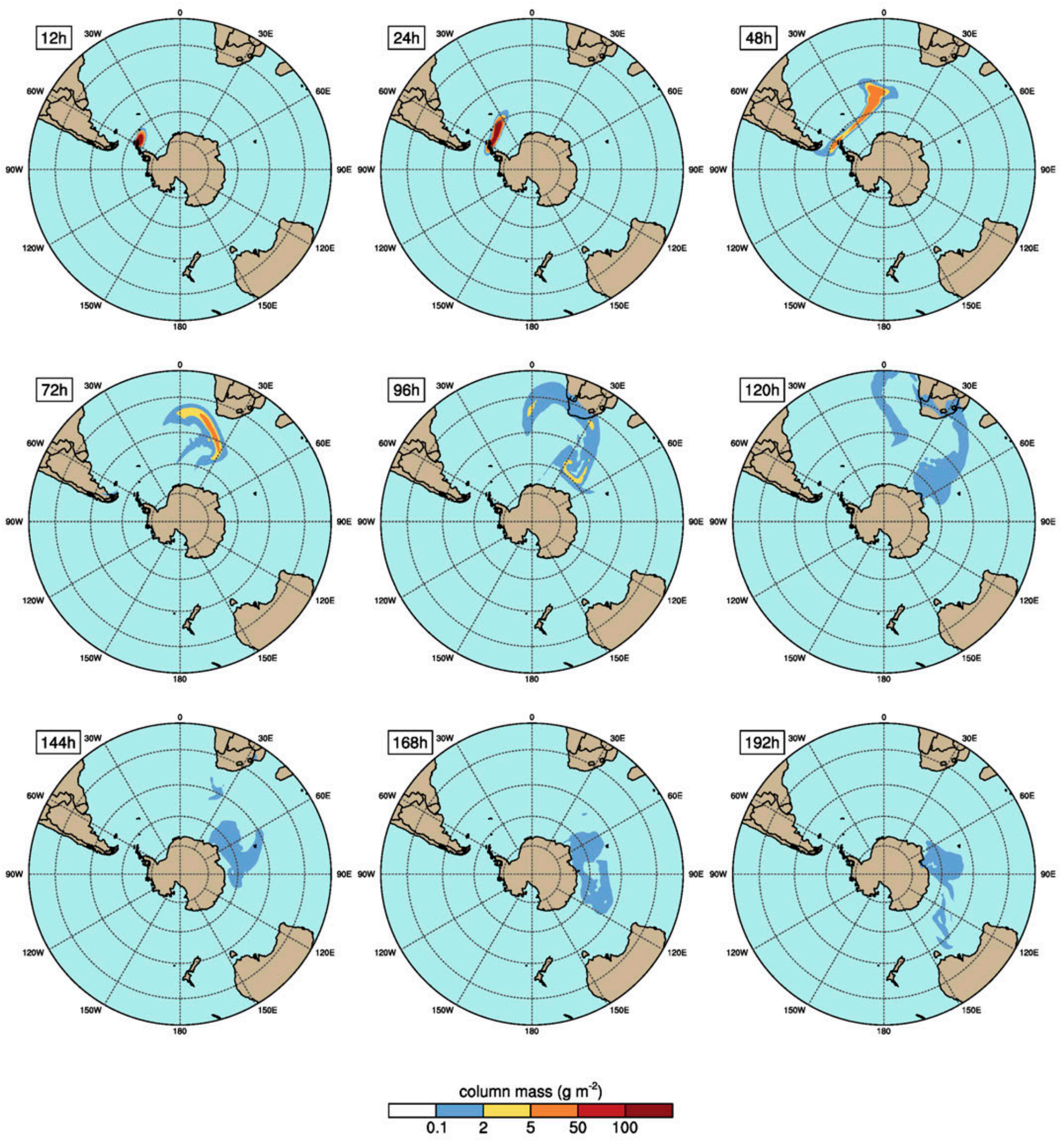

Figure S1.8. NMMB-MONARCH-ASH total ash column mass load (in g m^{-2}) during the Austral summer period at different time instants after the eruption start. Simulation considering an eruption column height of 10 km. This figure was generated with NCAR Command Language (NCL) version 6.1.2 (Boulder, Colorado: UCAR/NCAR/CISL/TDD. http://dx.doi.org/10.5065/D6WD3XH5). Final layout was achieved using Adobe Illustrator CC 2015.3.1 (Copyright © 1987–2016 Adobe Systems Incorporated and its licensors).

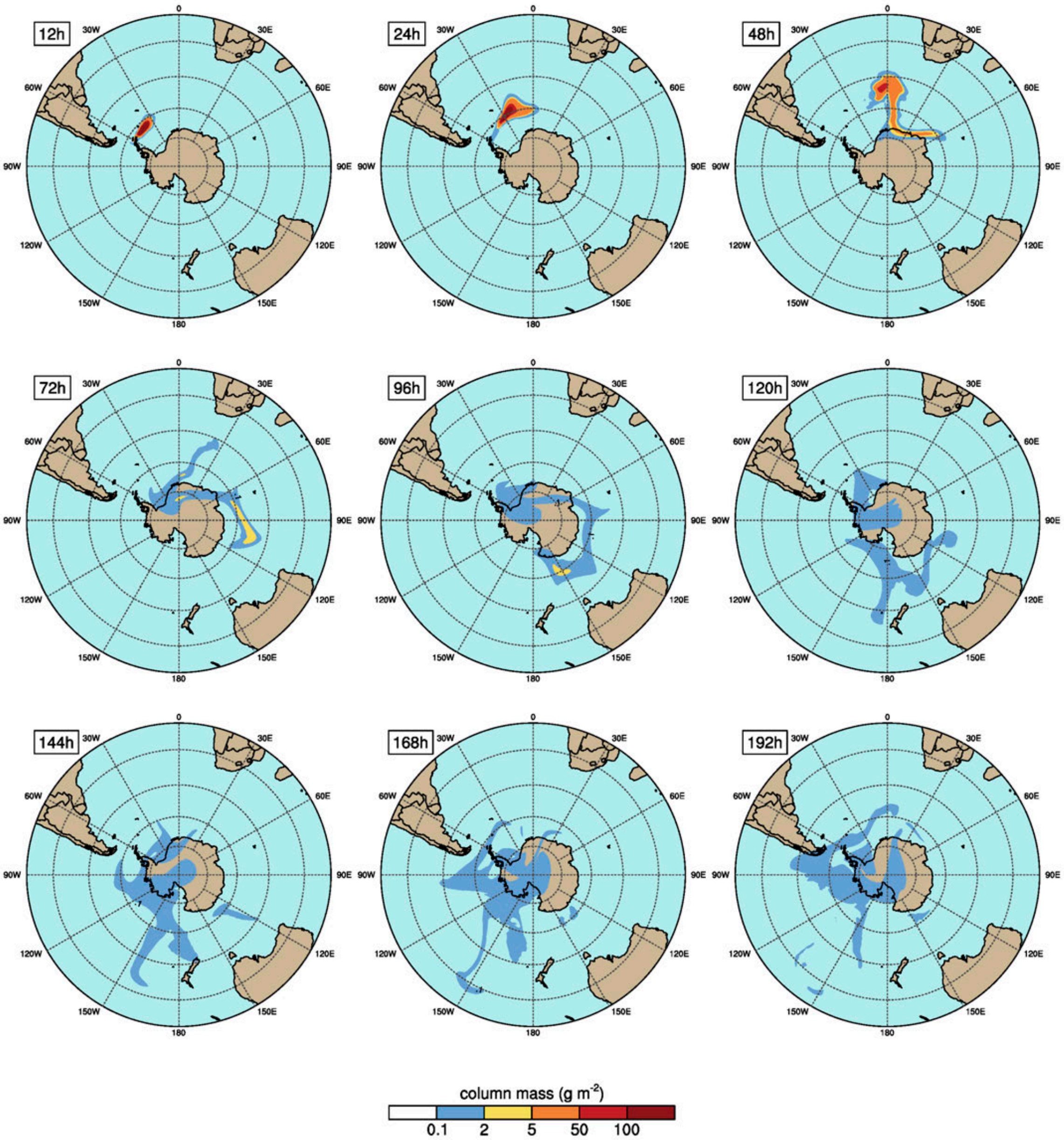

Figure S1.9. NMMB-MONARCH-ASH total ash column mass load (in g m^{-2}) during the Austral summer period at different time instants after the eruption start. Simulation considering an eruption column height of 15 km. This figure was generated with NCAR Command Language (NCL) version 6.1.2 (Boulder, Colorado: UCAR/NCAR/CISL/TDD. http://dx.doi.org/10.5065/D6WD3XH5). Final layout was achieved using Adobe Illustrator CC 2015.3.1 (Copyright © 1987–2016 Adobe Systems Incorporated and its licensors).

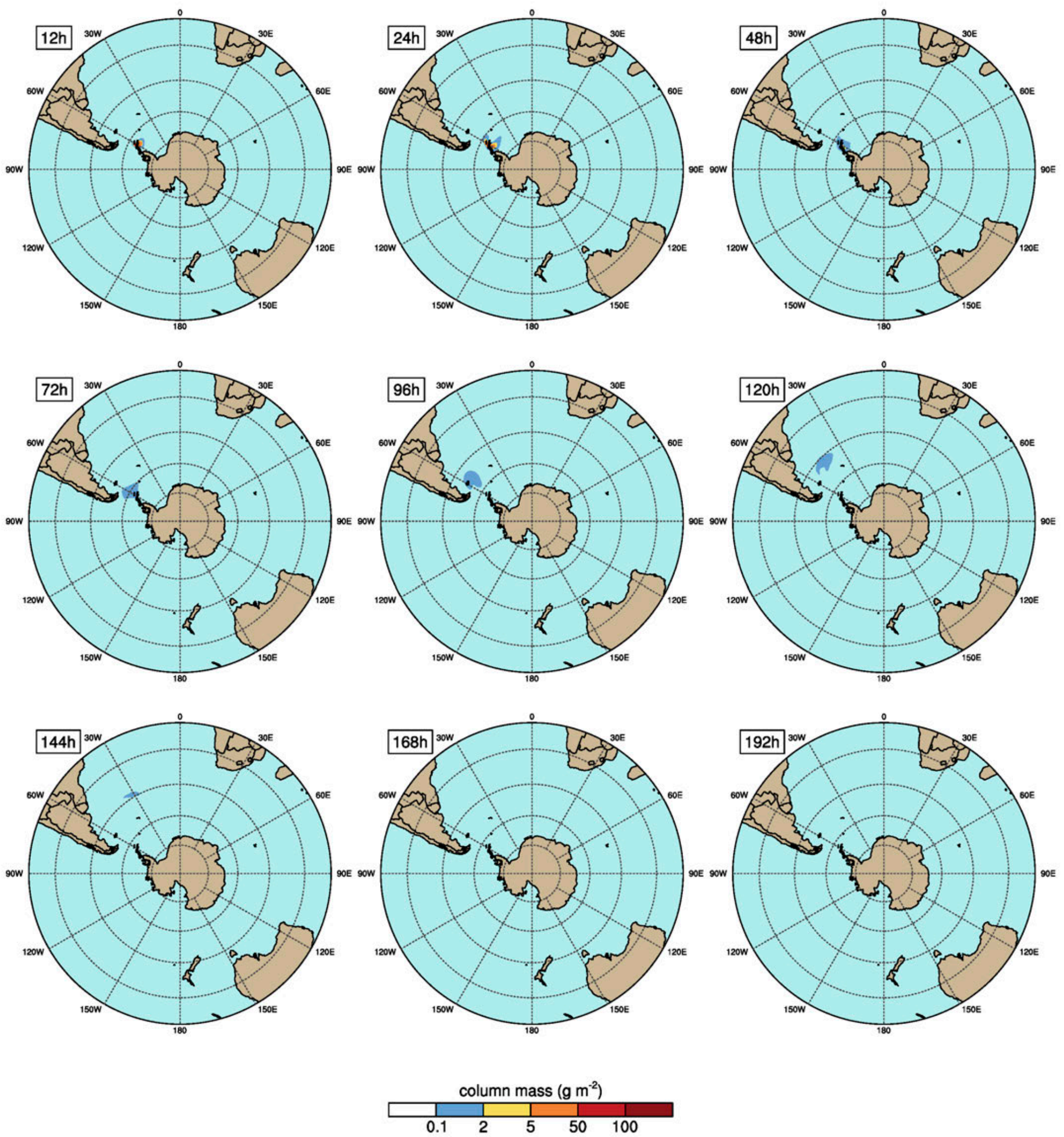

Figure S1.10. NMMB-MONARCH-ASH total ash column mass load (in g m^{-2}) during the Austral winter period at different time instants after the eruption start. Simulation considering an eruption column height of 5 km. This figure was generated with NCAR Command Language (NCL) version 6.1.2 (Boulder, Colorado: UCAR/NCAR/CISL/TDD. http://dx.doi.org/10.5065/D6WD3XH5). Final layout was achieved using Adobe Illustrator CC 2015.3.1 (Copyright © 1987–2016 Adobe Systems Incorporated and its licensors).

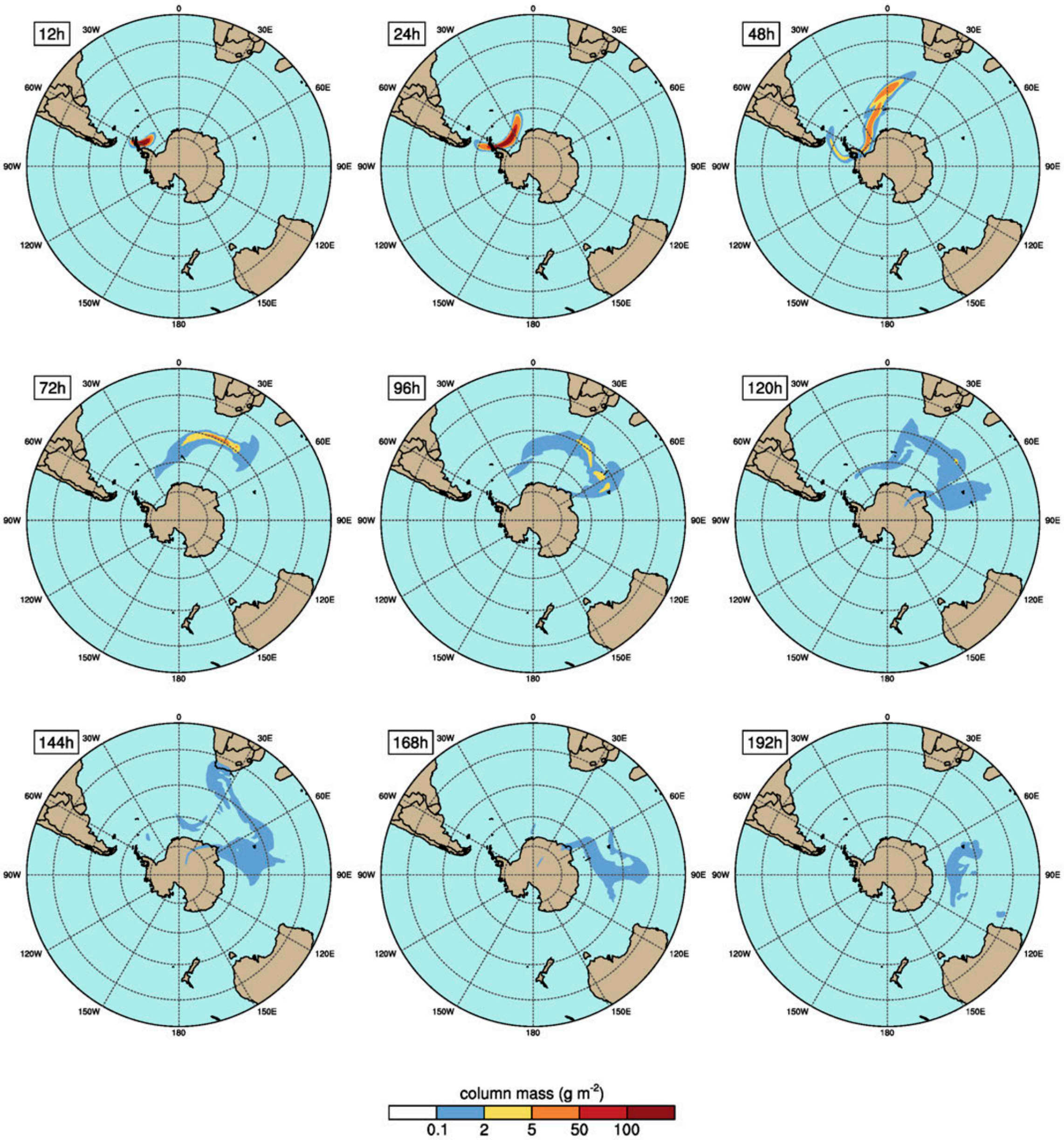

Figure S1.11. NMMB-MONARCH-ASH total ash column mass load (in g m^{-2}) during the Austral winter period at different time instants after the eruption start. Simulation considering an eruption column height of 10 km. This figure was generated with NCAR Command Language (NCL) version 6.1.2 (Boulder, Colorado: UCAR/NCAR/CISL/TDD. http://dx.doi.org/10.5065/D6WD3XH5). Final layout was achieved using Adobe Illustrator CC 2015.3.1 (Copyright © 1987–2016 Adobe Systems Incorporated and its licensors).

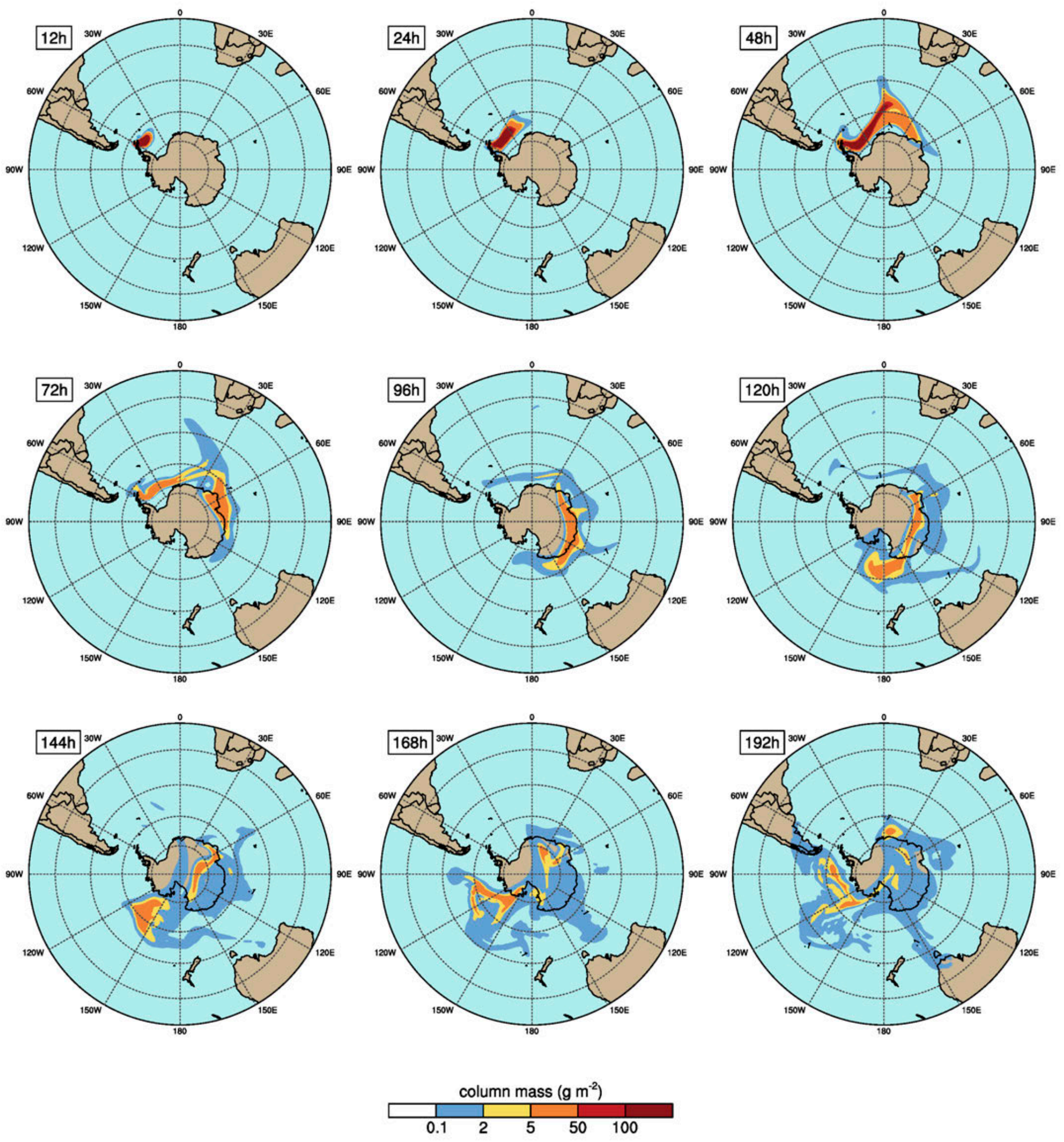

Figure S1.12. NMMB-MONARCH-ASH total ash column mass load (in g m^{-2}) during the Austral winter period at different time instants after the eruption start. Simulation considering an eruption column height of 15 km. This figure was generated with NCAR Command Language (NCL) version 6.1.2 (Boulder, Colorado: UCAR/NCAR/CISL/TDD. http://dx.doi.org/10.5065/D6WD3XH5). Final layout was achieved using Adobe Illustrator CC 2015.3.1 (Copyright © 1987–2016 Adobe Systems Incorporated and its licensors).

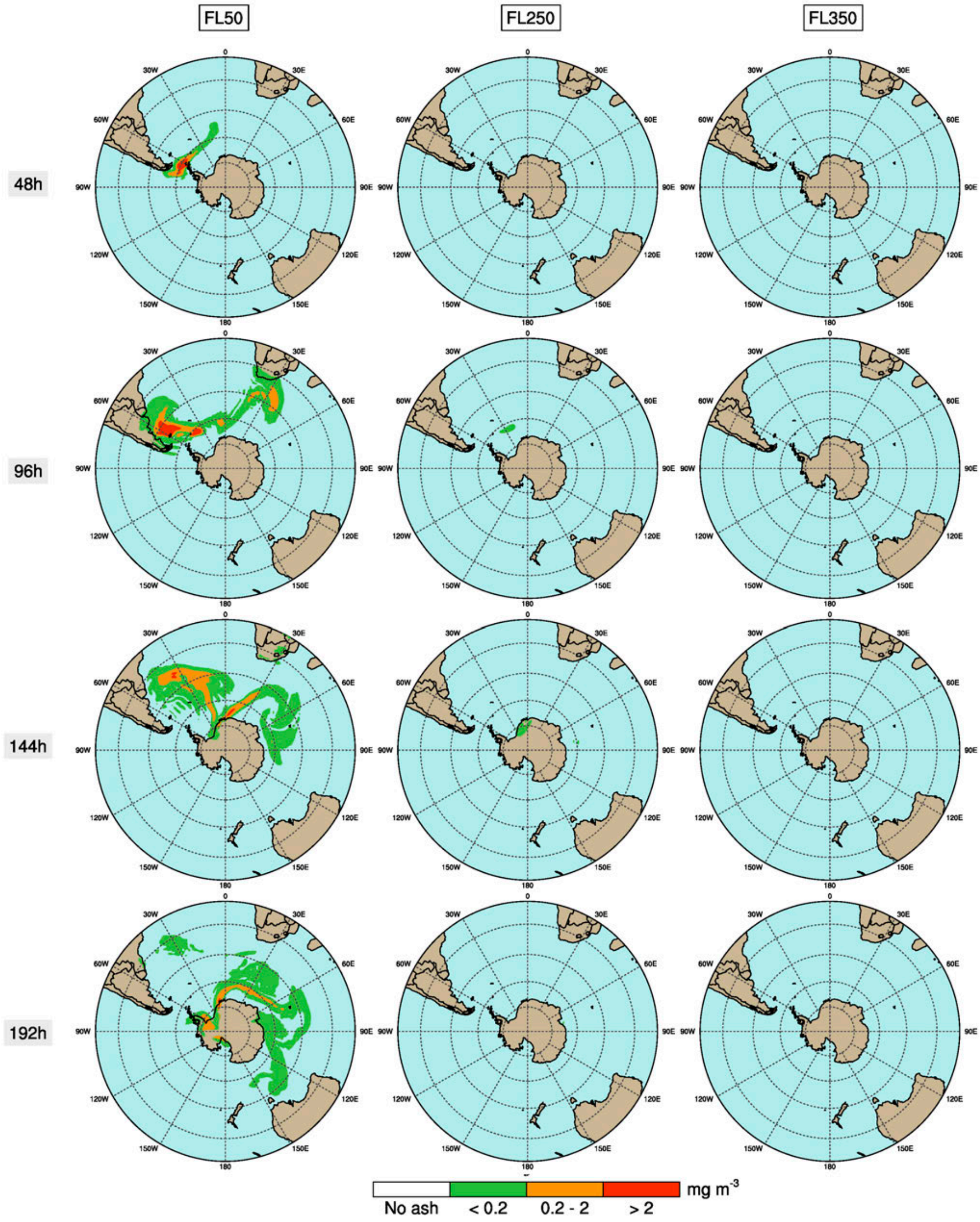

Figure S1.13. NMMB-MONARCH-ASH Flight Level ash concentrations (in mg m^{-3}) at FL50 (left), FL250 (middle), and FL350 (right) at different time slices during the Austral summer period. Safe ash concentration thresholds are shown (red contours illustrate “No Flying” zones). Simulation considering an eruption column height of 5 km. This figure was generated with NCAR Command Language (NCL) version 6.1.2 (Boulder, Colorado: UCAR/NCAR/CISL/TDD. http://dx.doi.org/10.5065/D6WD3XH5). Final layout was achieved using Adobe Illustrator CC 2015.3.1 (Copyright © 1987–2016 Adobe Systems Incorporated and its licensors).

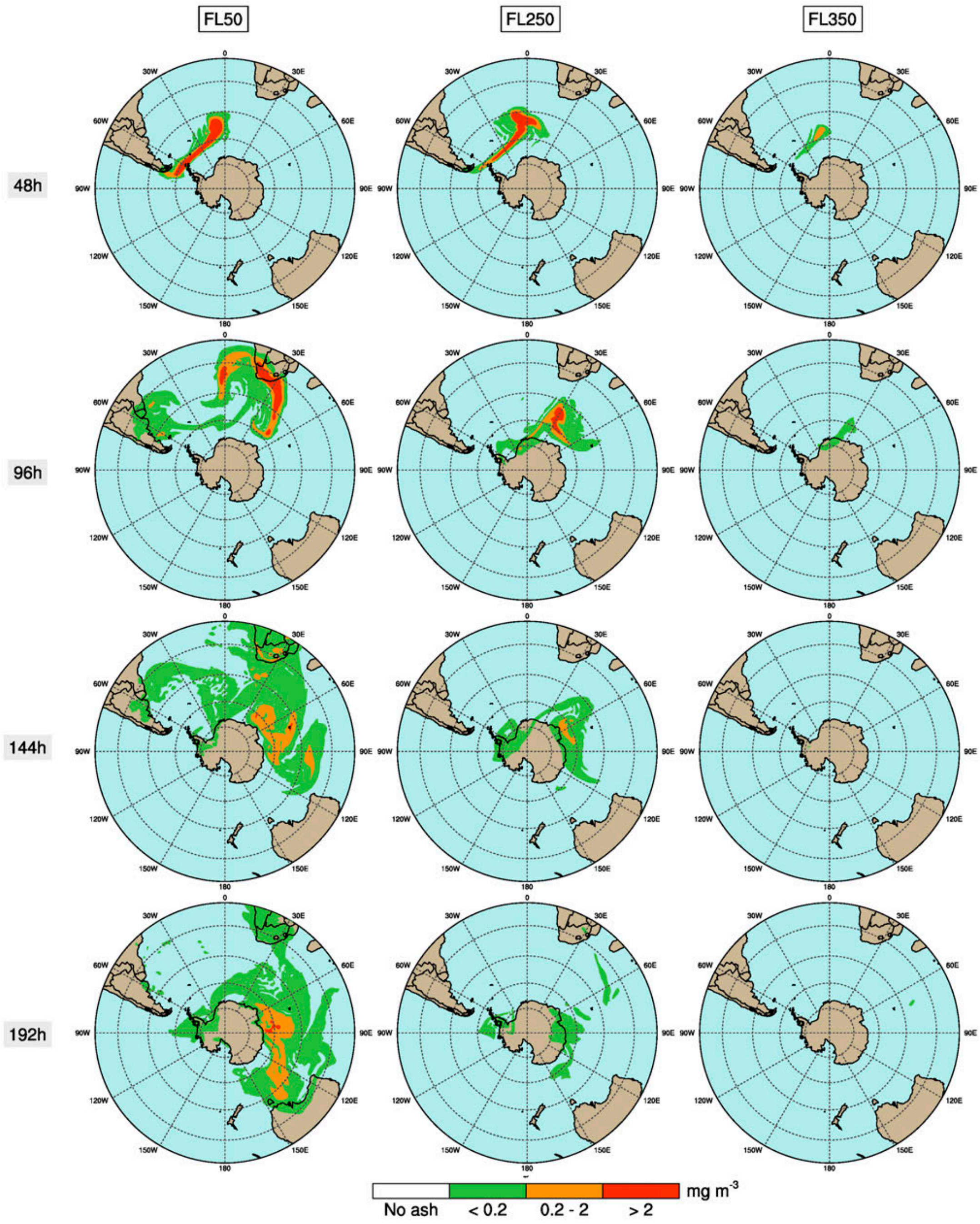

Figure S1.14. NMMB-MONARCH-ASH Flight Level ash concentrations (in mg m^{-3}) at FL50 (left), FL250 (middle), and FL350 (right) at different time slices during the Austral summer period. Safe ash concentration thresholds are shown (red contours illustrate "No Flying" zones). Simulation considering an eruption column height of 10 km. This figure was generated with NCAR Command Language (NCL) version 6.1.2 (Boulder, Colorado: UCAR/NCAR/CISL/TDD. http://dx.doi.org/10.5065/D6WD3XH5). Final layout was achieved using Adobe Illustrator CC 2015.3.1 (Copyright © 1987–2016 Adobe Systems Incorporated and its licensors).

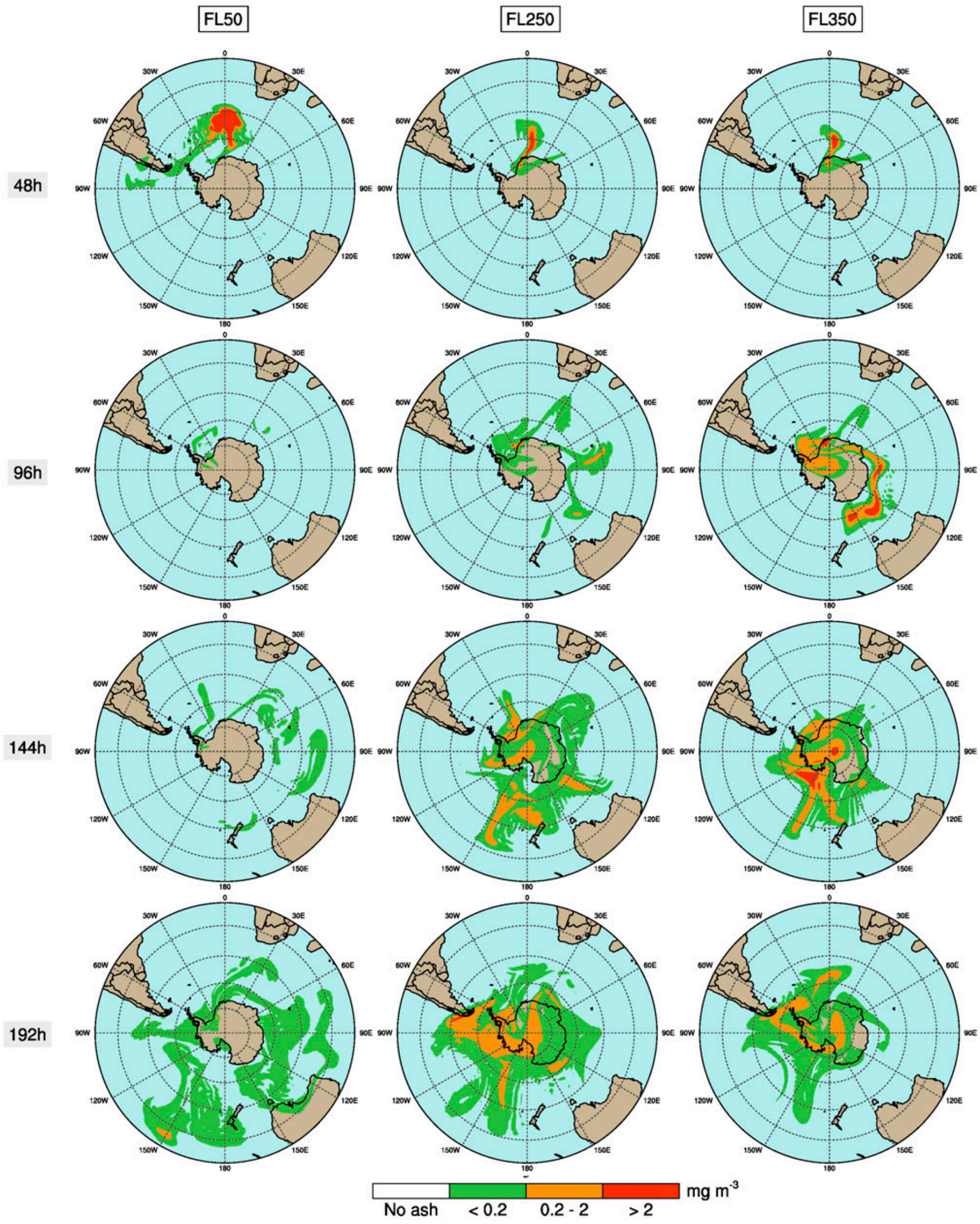

Figure S1.15. NMMB-MONARCH-ASH Flight Level ash concentrations (in mg m^{-3}) at FL50 (left), FL250 (middle), and FL350 (right) at different time slices during the Austral summer period. Safe ash concentration thresholds are shown (red contours illustrate "No Flying" zones). Simulation considering an eruption column height of 15 km. This figure was generated with NCAR Command Language (NCL) version 6.1.2 (Boulder, Colorado: UCAR/NCAR/CISL/TDD. http://dx.doi.org/10.5065/D6WD3XH5). Final layout was achieved using Adobe Illustrator CC 2015.3.1 (Copyright © 1987–2016 Adobe Systems Incorporated and its licensors).

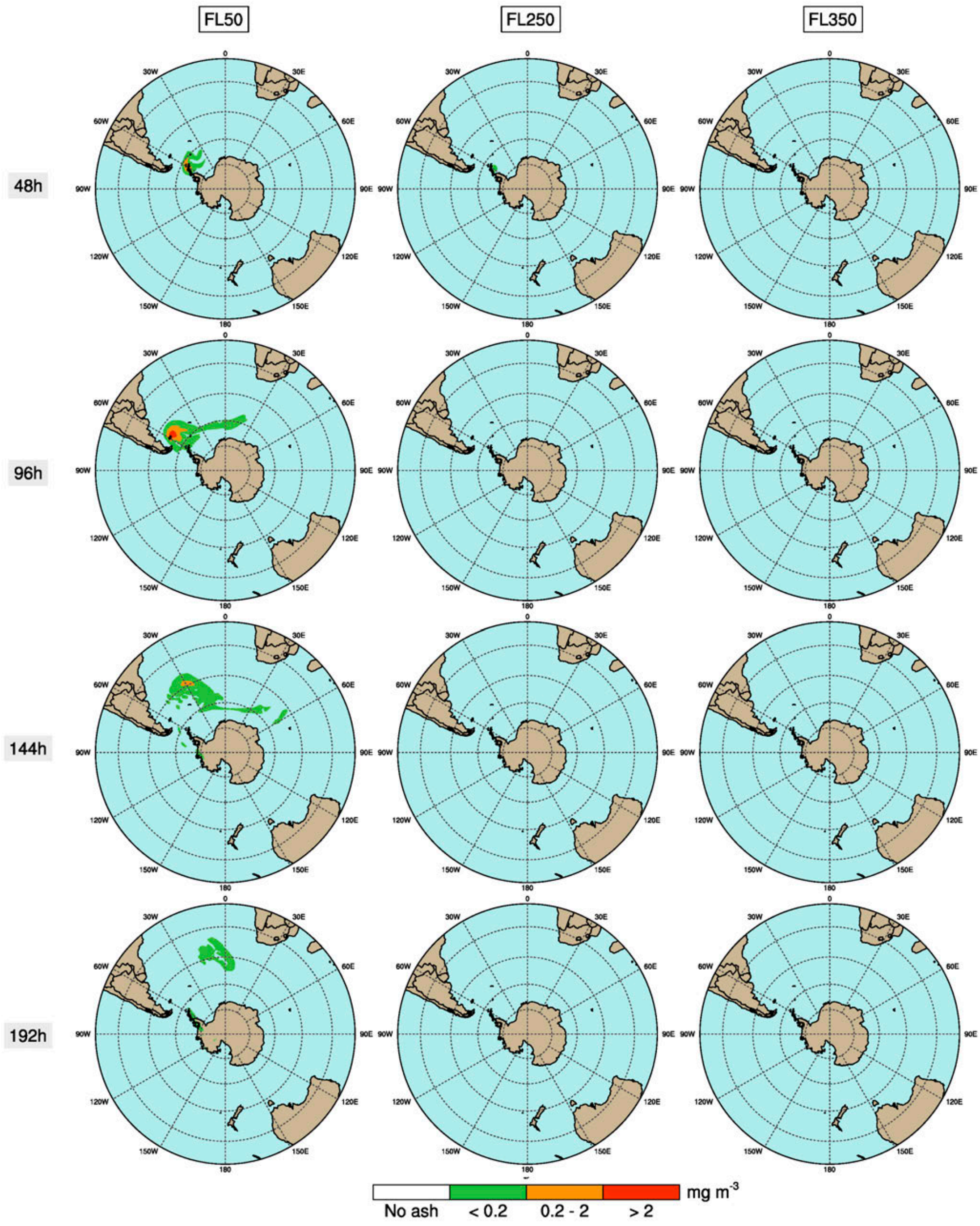

Figure S1.16. NMMB-MONARCH-ASH Flight Level ash concentrations (in mg m^{-3}) at FL50 (left), FL250 (middle), and FL350 (right) at different time slices during the Austral winter period. Safe ash concentration thresholds are shown (red contours illustrate “No Flying” zones). Simulation considering an eruption column height of 5 km. This figure was generated with NCAR Command Language (NCL) version 6.1.2 (Boulder, Colorado: UCAR/NCAR/CISL/TDD. http://dx.doi.org/10.5065/D6WD3XH5). Final layout was achieved using Adobe Illustrator CC 2015.3.1 (Copyright © 1987–2016 Adobe Systems Incorporated and its licensors).

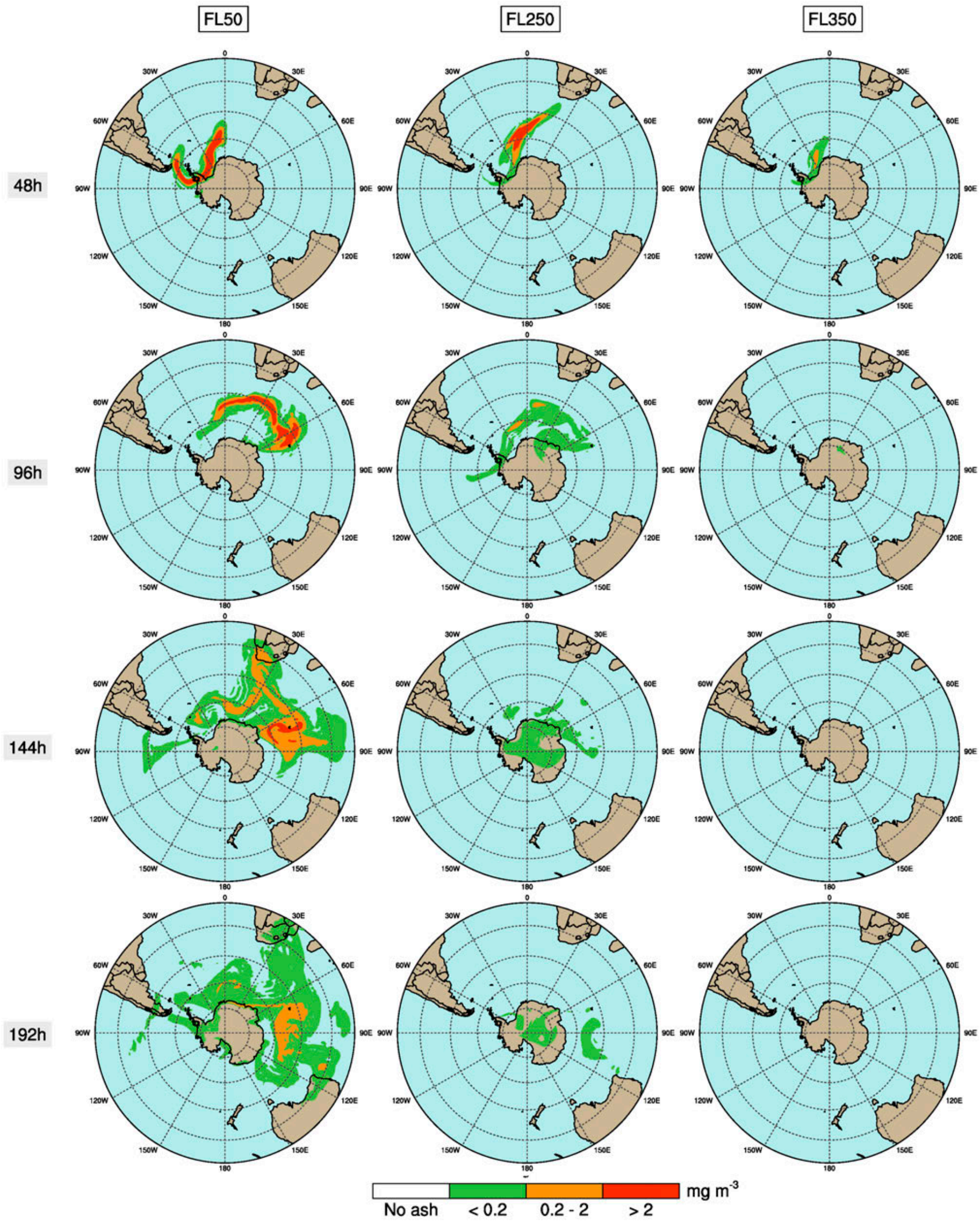

Figure S1.17. NMMB-MONARCH-ASH Flight Level ash concentrations (in mg m^{-3}) at FL50 (left), FL250 (middle), and FL350 (right) at different time slices during the Austral winter period. Safe ash concentration thresholds are shown (red contours illustrate "No Flying" zones). Simulation considering an eruption column height of 10 km. This figure was generated with NCAR Command Language (NCL) version 6.1.2 (Boulder, Colorado: UCAR/NCAR/CISL/TDD. http://dx.doi.org/10.5065/D6WD3XH5). Final layout was achieved using Adobe Illustrator CC 2015.3.1 (Copyright © 1987–2016 Adobe Systems Incorporated and its licensors).

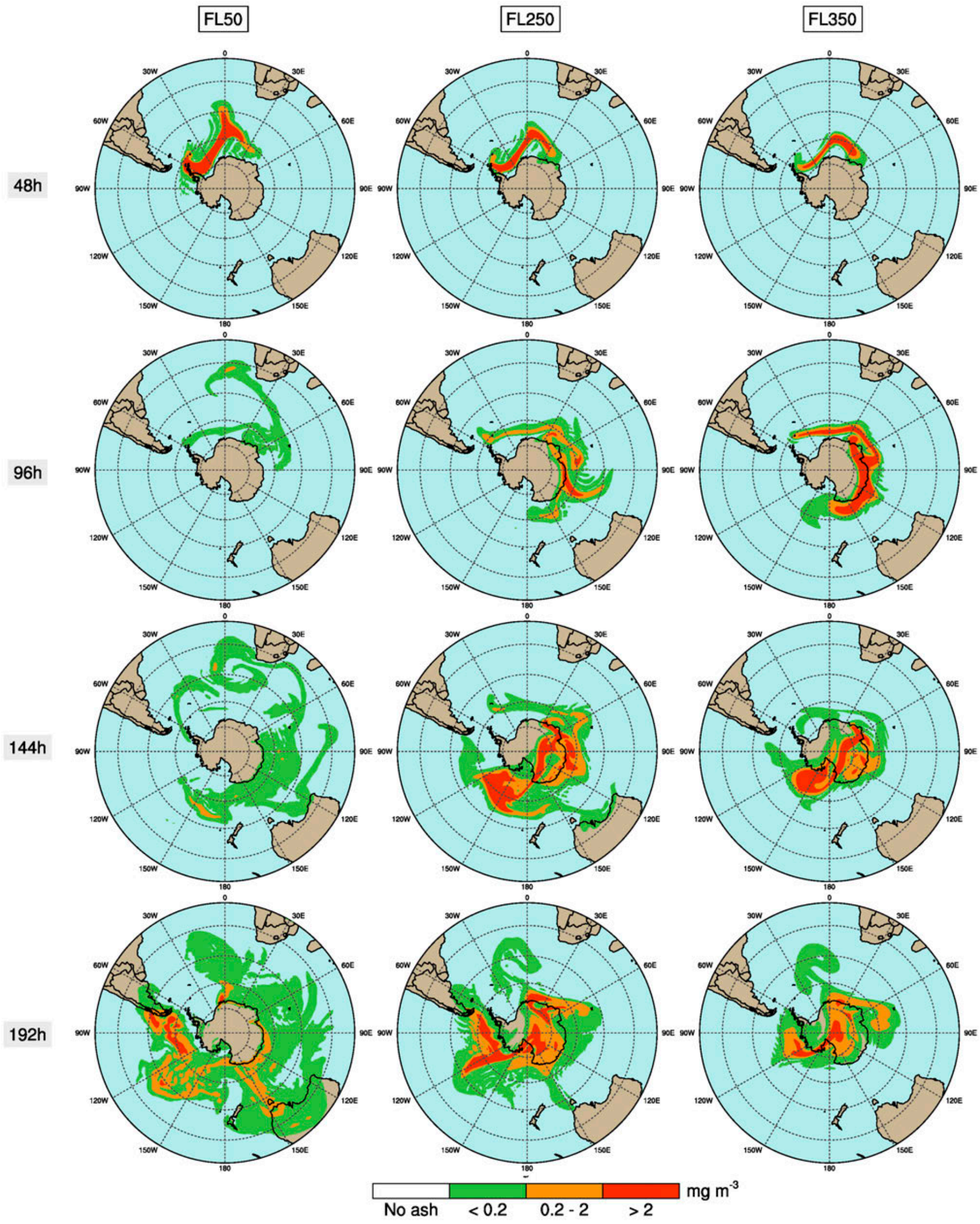

Figure S1.18. NMMB-MONARCH-ASH Flight Level ash concentrations (in mg m^{-3}) at FL50 (left), FL250 (middle), and FL350 (right) at different time slices during the Austral winter period. Safe ash concentration thresholds are shown (red contours illustrate "No Flying" zones). Simulation considering an eruption column height of 15 km. This figure was generated with NCAR Command Language (NCL) version 6.1.2 (Boulder, Colorado: UCAR/NCAR/CISL/TDD. http://dx.doi.org/10.5065/D6WD3XH5). Final layout was achieved using Adobe Illustrator CC 2015.3.1 (Copyright © 1987–2016 Adobe Systems Incorporated and its licensors).

Potential ash impact from Antarctic volcanoes:

Insights from Deception Island's most recent eruption

A. Geyer[(1)], A. Martí[(2)], S. Giralt[(1)], A. Folch[(2)]

(1) Institute of Earth Sciences Jaume Almera (ICTJA-CSIC), Lluis Solé i Sabaris s/n, 08028 Barcelona, Spain

(2) Barcelona Supercomputing Center (BSC), Jordi Girona 29, 08034 Barcelona, Spain

SUPPLEMENTARY MATERIAL 2

Summer 1992 / Summer 1999

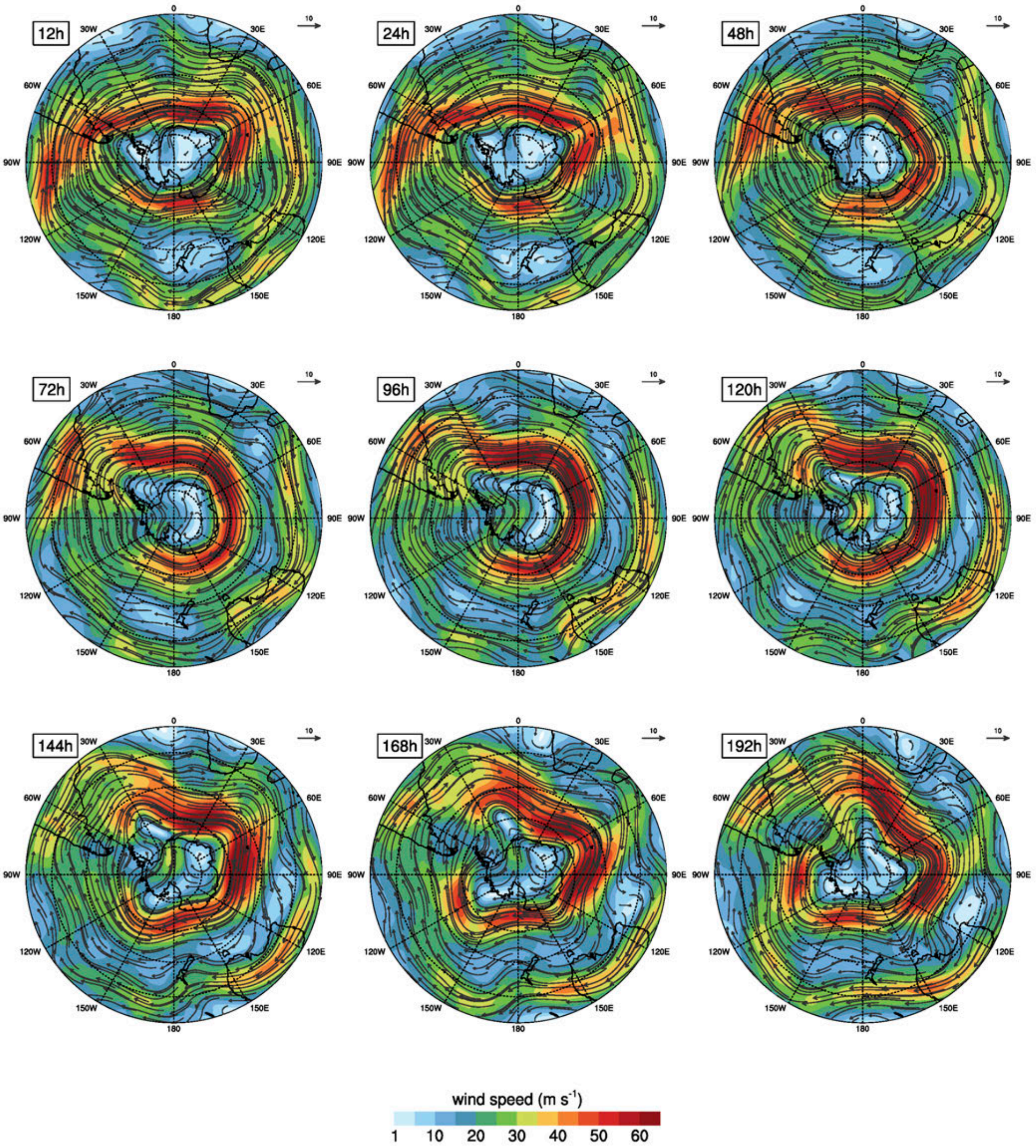

Figure S2.1. NMMB-MONARCH-ASH meteorological model results over the South Pole during the Austral summer period (1992). Plots show wind vectors and velocity contours (in ms^{-1}) at 5 km height during 8 days (192h). This figure was generated with NCAR Command Language (NCL) version 6.1.2 (Boulder, Colorado: UCAR/NCAR/CISL/TDD. http://dx.doi.org/10.5065/D6WD3XH5). Final layout was achieved using Adobe Illustrator CC 2015.3.1 (Copyright © 1987–2016 Adobe Systems Incorporated and its licensors).

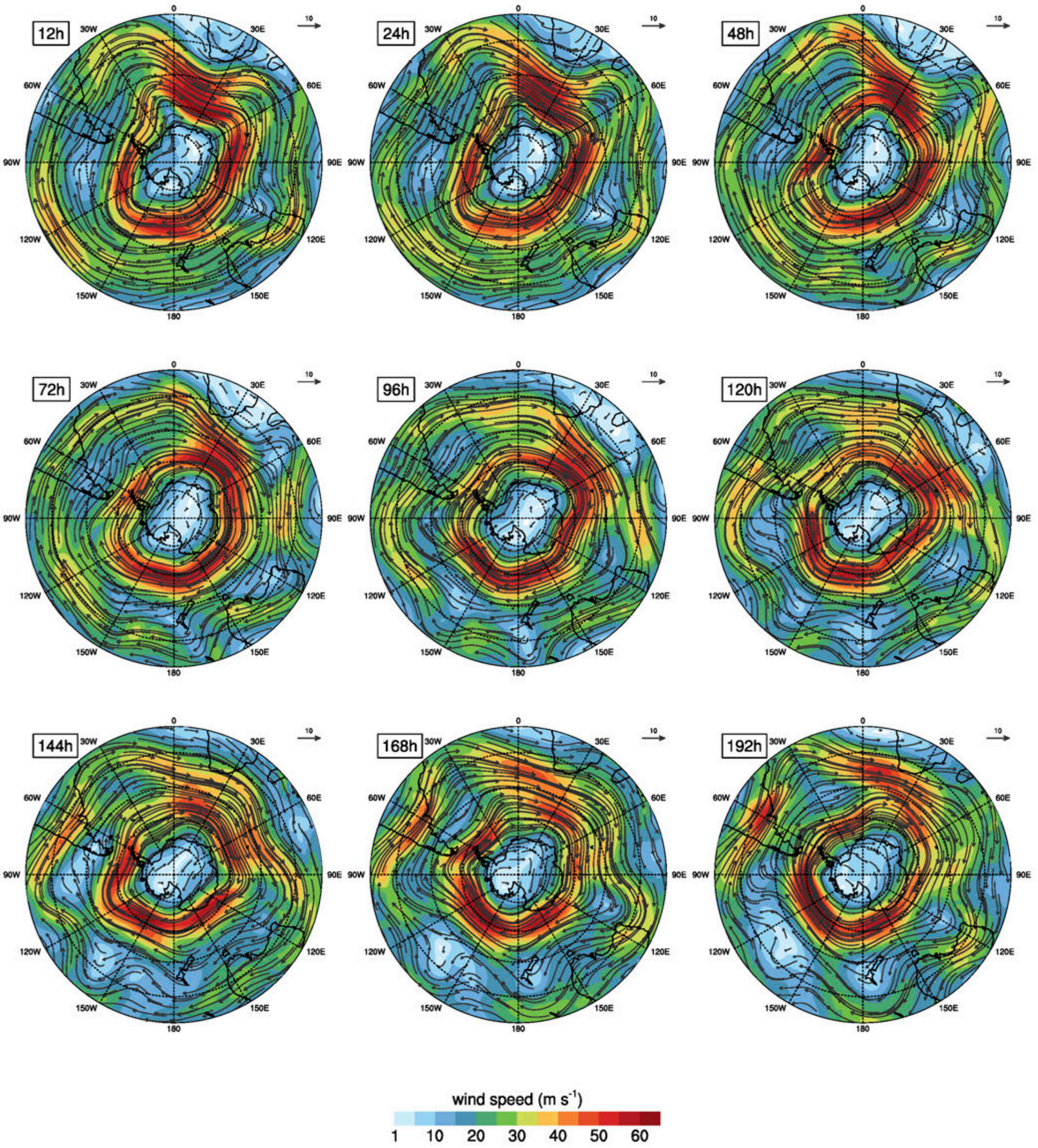

Figure S2.2. NMMB-MONARCH-ASH meteorological model results over the South Pole during the Austral summer period (1999). Plots show wind vectors and velocity contours (in ms^{-1}) at 5 km height during 8 days (192h). This figure was generated with NCAR Command Language (NCL) version 6.1.2 (Boulder, Colorado: UCAR/NCAR/CISL/TDD. http://dx.doi.org/10.5065/D6WD3XH5). Final layout was achieved using Adobe Illustrator CC 2015.3.1 (Copyright © 1987–2016 Adobe Systems Incorporated and its licensors).

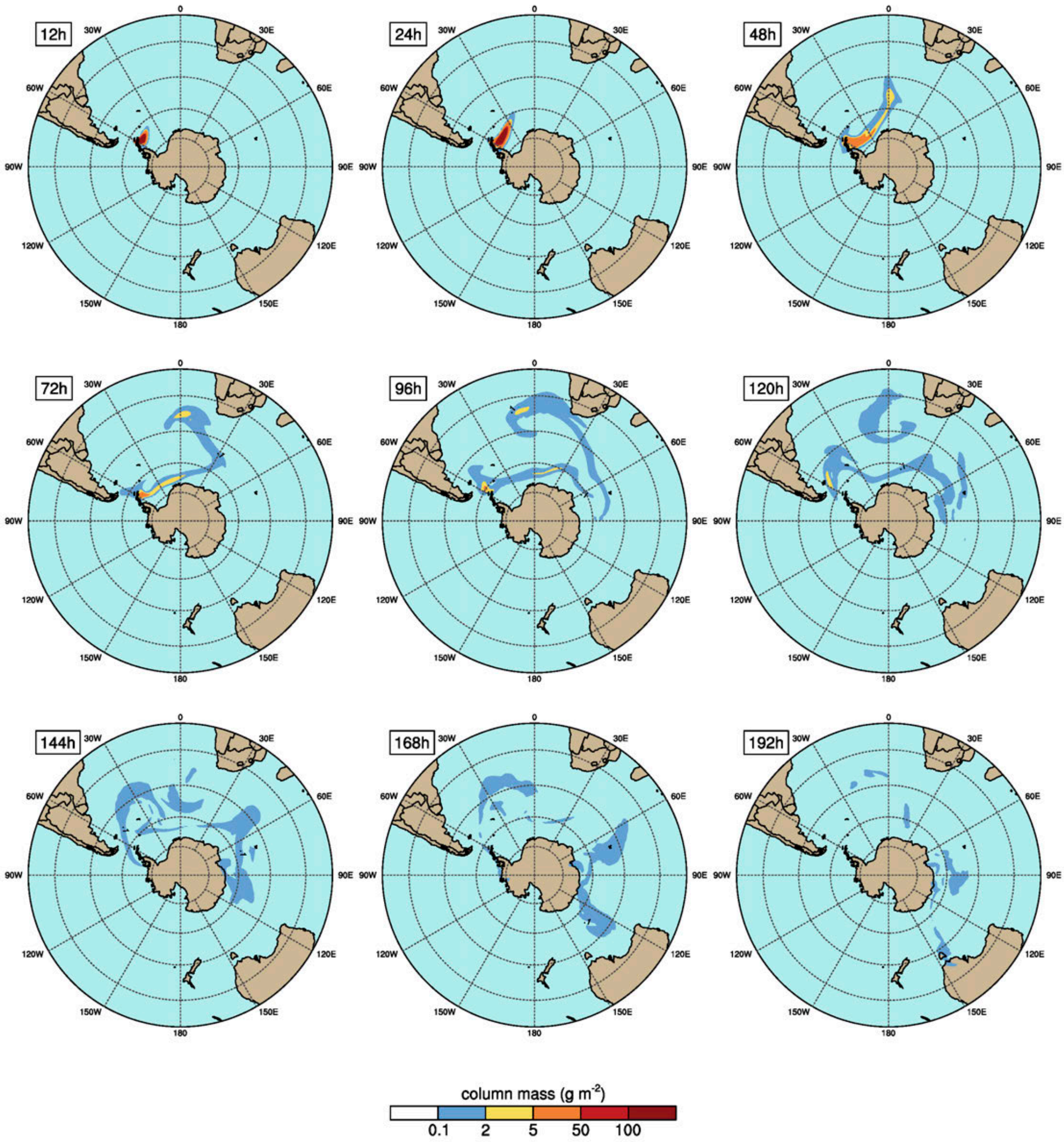

Figure S2.3. NMMB-MONARCH-ASH total ash column mass load (in g m^{-2}) during the Austral summer period (1992) at different time instants after the eruption start. Simulation considering an eruption column height of 10 km. This figure was generated with NCAR Command Language (NCL) version 6.1.2 (Boulder, Colorado: UCAR/NCAR/CISL/TDD. http://dx.doi.org/10.5065/D6WD3XH5). Final layout was achieved using Adobe Illustrator CC 2015.3.1 (Copyright © 1987–2016 Adobe Systems Incorporated and its licensors).

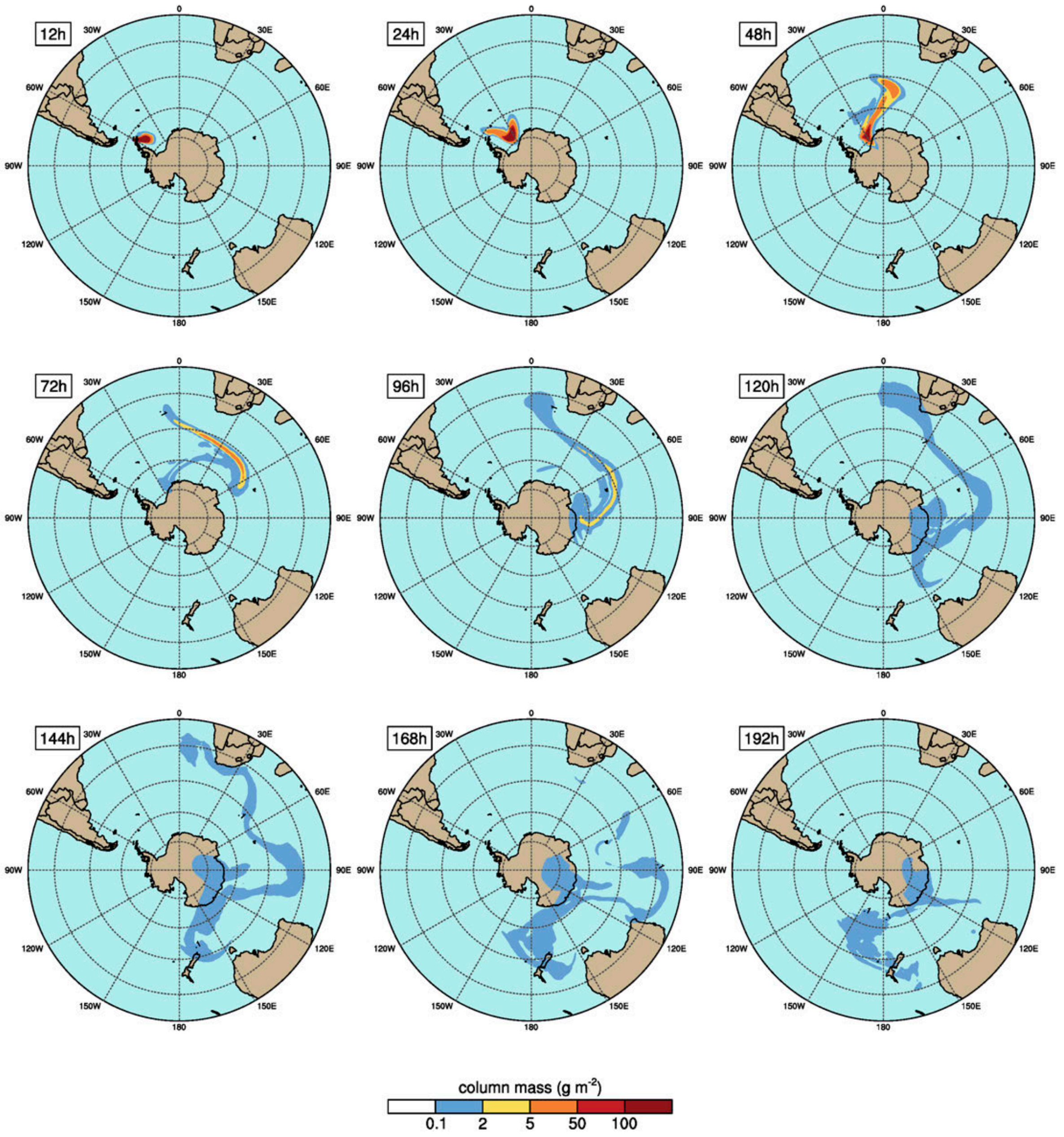

Figure S2.4. NMMB-MONARCH-ASH total ash column mass load (in g m^{-2}) during the Austral summer period (1999) at different time instants after the eruption start. Simulation considering an eruption column height of 10 km. The yellow star indicates the location of Deception Island. This figure was generated with NCAR Command Language (NCL) version 6.1.2 (Boulder, Colorado: UCAR/NCAR/CISL/TDD. http://dx.doi.org/10.5065/D6WD3XH5). Final layout was achieved using Adobe Illustrator CC 2015.3.1 (Copyright © 1987–2016 Adobe Systems Incorporated and its licensors).

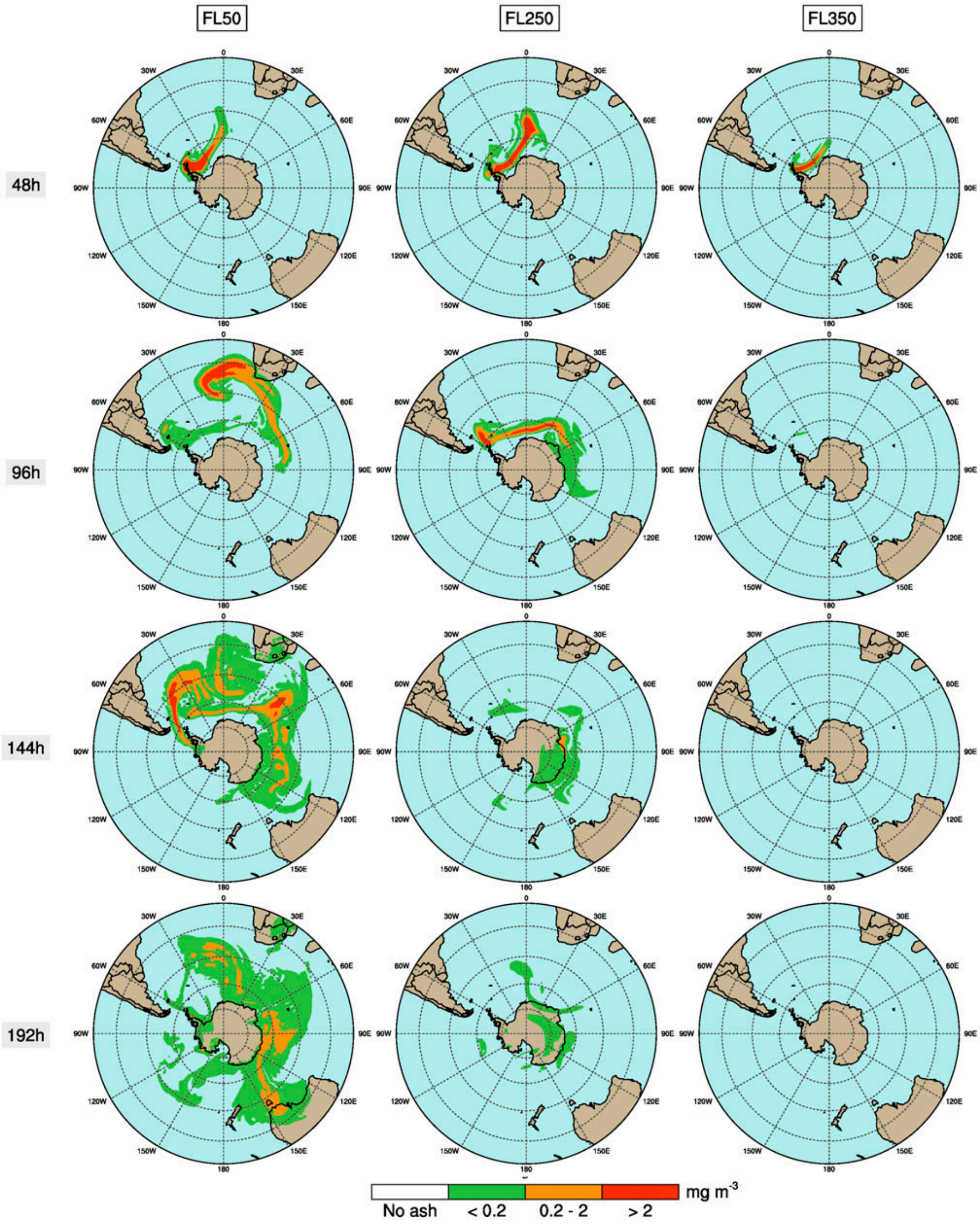

Figure S2.5. NMMB-MONARCH-ASH Flight Level ash concentrations (in mg m^{-3}) at FL50 (left), FL250 (middle), and FL350 (right) at different time slices during the summer period (1992). Safe ash concentration thresholds are shown (red contours illustrate "No Flying" zones). Simulation considering an eruption column height of 10 km. This figure was generated with NCAR Command Language (NCL) version 6.1.2 (Boulder, Colorado: UCAR/NCAR/CISL/TDD. http://dx.doi.org/10.5065/D6WD3XH5). Final layout was achieved using Adobe Illustrator CC 2015.3.1 (Copyright © 1987–2016 Adobe Systems Incorporated and its licensors).

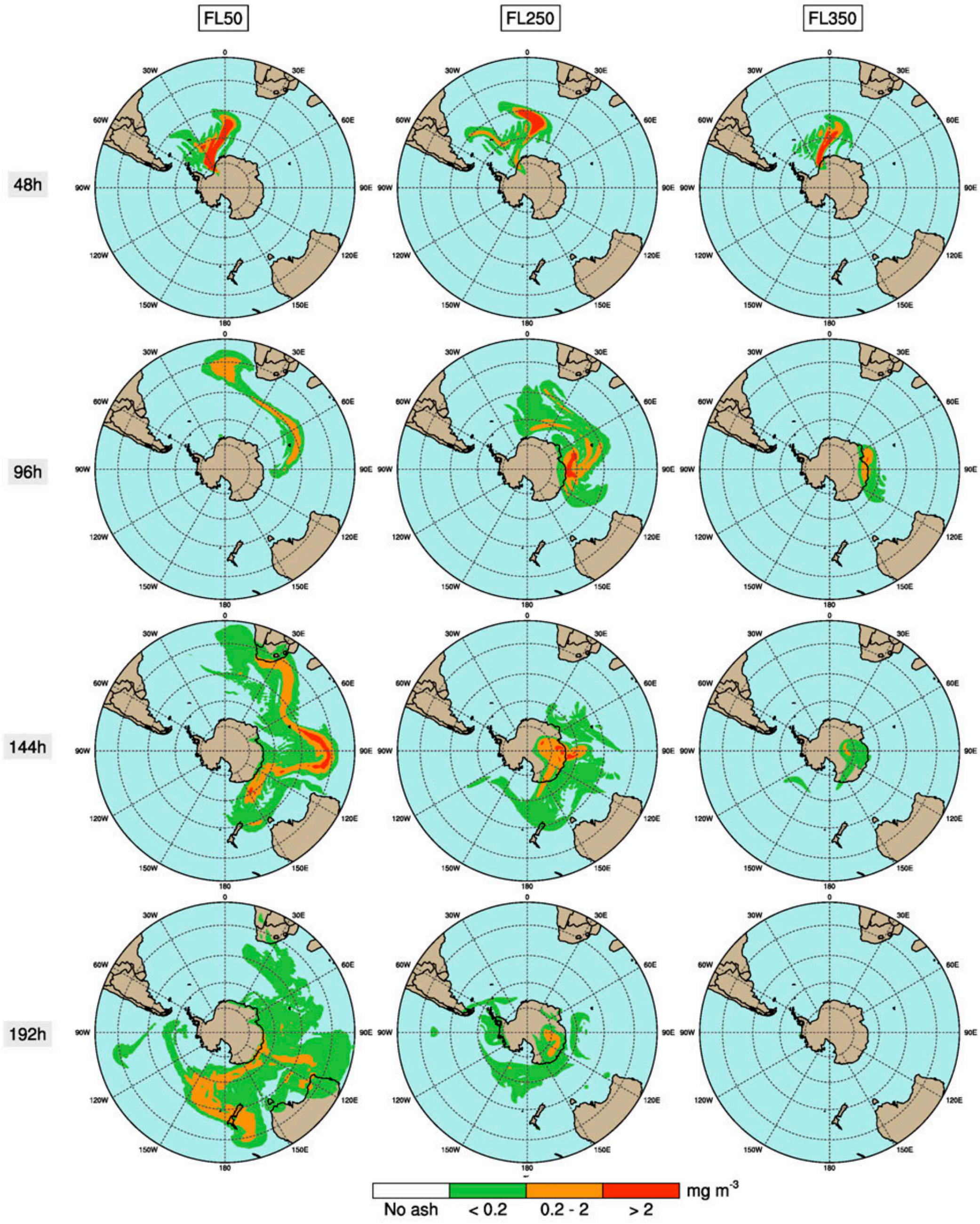

Figure S2.6. NMMB-MONARCH-ASH Flight Level ash concentrations (in mg m^{-3}) at FL50 (left), FL250 (middle), and FL350 (right) at different time slices during the summer period (1999). Safe ash concentration thresholds are shown (red contours illustrate "No Flying" zones). Simulation considering an eruption column height of 10 km. This figure was generated with NCAR Command Language (NCL) version 6.1.2 (Boulder, Colorado: UCAR/NCAR/CISL/TDD. http://dx.doi.org/10.5065/D6WD3XH5). Final layout was achieved using Adobe Illustrator CC 2015.3.1 (Copyright © 1987–2016 Adobe Systems Incorporated and its licensors).

Potential ash impact from Antarctic volcanoes:

Insights from Deception Island's most recent eruption

A. Geyer[(1)], A. Martí[(2)], S. Giralt[(1)], A. Folch[(2)]

(1) Institute of Earth Sciences Jaume Almera (ICTJA-CSIC), Lluis Solé i Sabaris s/n, 08028 Barcelona, Spain

(2) Barcelona Supercomputing Center (BSC), Jordi Girona 29, 08034 Barcelona, Spain

SUPPLEMENTARY MATERIAL 3

Videos

Video 1: NMMB-MONARCH-ASH total ash column mass load (in g m^{-2}) during the Austral summer period at different time instants after the eruption start. Simulation considering an eruption column height of 5 km. The figure was generated with NCAR Command Language (NCL) version 6.1.2 (Boulder, Colorado: UCAR/NCAR/CISL/TDD. http://dx.doi.org/10.5065/D6WD3XH5).

Video 2: NMMB-MONARCH-ASH total ash column mass load (in g m^{-2}) during the Austral summer period at different time instants after the eruption start. Simulation considering an eruption column height of 10 km. The figure was generated with NCAR Command Language (NCL) version 6.1.2 (Boulder, Colorado: UCAR/NCAR/CISL/TDD. http://dx.doi.org/10.5065/D6WD3XH5).

Video 3: NMMB-MONARCH-ASH total ash column mass load (in g m^{-2}) during the Austral summer period at different time instants after the eruption start. Simulation considering an eruption column height of 15 km. The figure was generated with NCAR Command Language (NCL) version 6.1.2 (Boulder, Colorado: UCAR/NCAR/CISL/TDD. http://dx.doi.org/10.5065/D6WD3XH5).
